# Convergent evolution in the late Permian megaphyllous leaves of the Noeggerathiales progymnosperm *Paratingia* and the cycad *Plagiozamites*

**DOI:** 10.1093/aob/mcaf272

**Published:** 2025-10-29

**Authors:** Yi-Fei Qin, Xiao-Yuan He, Shi-Jun Wang, Xinshi Cheng, Jason Hilton, Gar W Rothwell

**Affiliations:** Institute of Palaeontology, Yunnan Key Laboratory for Palaeobiology, Yunnan University, Kunming 650500, China; Institute of Palaeontology, Yunnan Key Laboratory for Palaeobiology, Yunnan University, Kunming 650500, China; State Key Laboratory of Palaeobiology and Stratigraphy, Nanjing Institute of Geology and Palaeontology, Chinese Academy of Sciences, Nanjing 210008, China; State Key Laboratory of Systematic and Evolutionary Botany, Institute of Botany, Chinese Academy of Sciences, Beijing 100093, China; School of Geography, Earth and Environmental Sciences, University of Birmingham, Edgbaston, Birmingham B15 2TT, UK; School of Geography, Earth and Environmental Sciences, University of Birmingham, Edgbaston, Birmingham B15 2TT, UK; Birmingham Institute of Forest Research, University of Birmingham, Edgbaston, Birmingham B15 2TT, UK; Department of Environmental and Plant Biology, Ohio University, Athens, OH 45701, USA; Department of Botany and Plant Pathology, Oregon State University, Corvallis, OR 97331, USA

**Keywords:** Anatomy, epidermal structures, morphology, leaf, Xuanwei Formation, *Gigantopteris* flora, Permian–Triassic mass extinction

## Abstract

**Background and Aims:**

The late Permian Xuanwei Formation represents the last refuge of the Palaeozoic fern-dominated Cathaysian (*Gigantopteris*) flora before its demise in the Permian–Triassic mass extinction. It contains two Noeggerathiales progymnosperm fertile shoots, but unequivocal Noeggerathiales leaves have not been identified. Co-occurring once-pinnate megaphyllous leaves of *Plagiozamites oblongifolius*, previously interpreted as cycad gymnosperms from morphology and epidermal characters, have also been considered as possible Noeggerathiales fronds based on their distinctive Ω-shaped rachis bundles and four rows of pinnules comprising two rows of large pinnules and two rows of small pinnules. We investigate newly collected *P. oblongifolius* leaves from the Xuanwei Formation to determine their affinity, accurately characterize species composition, elucidate features to distinguish the leaves of Noeggerathiales and cycads, and consider convergent evolution in these two systematically diverse plant orders.

**Methods:**

We examined the morphology and anatomy of *Plagiozamites oblongifolius* leaves using the acetate peel technique, and transmitted light and scanning electron microscopy.

**Key Results:**

The leaves have two rows of large pinnules, two rows of small pinnules, and epidermal characters consistent with Noeggerathiales. From these we identify three new species of the Noeggerathiales genus *Paratingia*, extending their stratigraphic range to the late Permian. *Paratingia* leaves are distinct from *Plagiozamites* cycad leaves, of which *at least* two species co-occur in the Xuanwei Formation, which have two rows of pinnules and different epidermal characters.

**Conclusions:**

Results emphasize parallel evolution in leaf form and anatomy among Noeggerathiales progymnosperms and cycads, which, despite superficial resemblance, are distinguished through gross morphology, rachis anatomy and epidermal characters. Our findings highlight complexities in accurately identifying fossil species from incomplete data and help clarify species richness and extinction magnitude in Cathaysia through the Palaeozoic–Mesozoic transition.

## INTRODUCTION

Late Permian strata in south-west China contain abundant plant fossils that represent the last refuge of the Palaeozoic fern-dominated Cathaysian (*Gigantopteris*) flora before its demise in the Permian–Triassic mass extinction (Feng *et al.*, 2020; Xu *et al.*, 2022; Dal Corso *et al.*, 2022). In this region two kinds of Noeggerathiales progymnosperm fertile organs have been reported from the late Permian Xuanwei Formation: *Discinites* cf. *orientalis* Li *et al.* (Zhao *et al.*, 1980) and *Dorsalistachya quadrisegmentorum* Wang *et al.* (Wang *et al.,* 2017). However, indisputable Noeggerathiales leaves, such as species of *Conchophyllum* Schenk, *Noeggerathia* Sternburg, *Paratingia* Zhang, *Tingia* Schenk and *Yuania* Sze, are not known from the Xuanwei Formation. Instead, leaves of *Plagiozamites oblongifolius* Halle are commonly preserved (Zhao *et al.*, 1980; Gu et Zhi, 1974; Guo *et al.*, 1990; Feng *et al.*, 2017). Leaves of *P*. *oblongifolius* from the formation vary greatly in pinnule morphology and size, and most are different from the pinnule shape and size of the type specimens of *P. oblongifolius* from the early–middle Permian of North China (Halle, 1927); whether they can be assigned to *P. oblongifolius* and whether they belong to a single species have not been determined previously.

There also is disagreement about the affinity of *Plagiozamites oblongifolius* leaves from the Xuanwei Formation. Based on distinctive features of rachis anatomy (Guo *et al.*, 1990) or epidermal structures (Feng *et al.*, 2017), *P. oblongifolius* has been interpreted as belonging within the Cycadales, whereas Wang *et al.* (2017) considered them to be the vegetative leaves of the fertile noeggerathialean *Dorsalistachya quadrisegmentorum* based on rachis anatomy. The investigation by Guo *et al.* (1990) was based on features of the vascular tissue from the leaf rachis only and lacked information on cuticular anatomy, and the investigation by Feng *et al.* (2017) was based on cuticular evidence alone. This information, therefore, presents an enigma because it is uncertain whether leaves assigned to *P. oblongifolius* from the Xuanwei Formation were produced by more than one independent plant lineage or if they included more than one kind of morphologically similar plant that could be distinguished on features of their cellular anatomy, including rachis organization and epidermal structure.

In the current study, we refer to fossils of this type as the *Plagiozamites oblongifolius* complex because if leaves of *P. oblongifolius* were produced by Noeggerathiales progymnosperms and Cycadales gymnosperms, these data could imply either a previously unrecognized evolutionary relationship between noeggatherialeans and cycads, or evolutionary convergence in leaf morphology in response to similar driving forces. Moreover, if leaves assigned to *P. oblongifolius* represent more than one natural taxon of leaf, it is important to understand the structural details to identify distinctive features that could distinguish Noeggerathiales from Cycadales, and in so doing to potentially recognize additional species richness within the Xuanwei Formation. Such additions would also help to more accurately document species losses during the Permian–Triassic mass extinction (e.g. Feng *et al.*, 2020; Dal Corso *et al.*, 2022; Xu *et al.*, 2022). Clearly, fossils of *P. oblongifolius* require more detailed investigation to clarify these uncertainties.

Here we document newly recognized leaves conforming to the general features of *Plagiozamites oblongifolius* from the late Permian Xuanwei Formation in south-west China with well-preserved morphological, anatomical and epidermal features. In comparison with previously published materials, these new fossils allow us to recognize that leaves with the general organization of *P. oblongifolius* form a species complex based on convergent features produced by two systematically distinct plant lineages, and enable us to resolve the affinities and evolutionary significance of specimens assigned to the taxon through detailed investigations of plant morphology and anatomy.

## MATERIALS AND METHODS

For this study, several exceptionally well-preserved leaves of *Plagiozamites oblongifolius* were collected in blocks of volcanic tuff from mine spoil at Qingyun coal mine, Fuyuan County, eastern Yunnan Province, SW China, located on the South China tectonic plate. The tuff occurs in the Xuanwei Formation and was deposited as discrete sedimentary beds amongst typical coal-measures facies. The Xuanwei Formation was deposited in the Wuchiapingian to Changhsingian stages of the Permian Period, ∼260–252 million years ago (Seyfullah *et al.*, 2010; Neregato *et al.*, 2016). Each specimen is partially exposed on the surface of the rock and partially embedded in the tuff matrix. The specimens have cellular details preserved in calcium carbonate; permineralization occurred rapidly after deposition, before significant decay had occurred (Neregato *et al.*, 2016). This comparatively rare mode of preservation allows both morphological and anatomical information to be recovered from the same fossil specimen (He *et al.*, 2021; Qin *et al.*, 2024).

Blocks of tuff containing individual leaves were cut using a water-cooled rock saw with a diamond blade to reveal longitudinal and cross-sections. Cut surfaces were then prepared by the cellulose acetate peel method (Joy *et al.*, 1956; Galtier and Phillips, 1999). Photography was undertaken using an Axio Imager A2 and a Nikon D3X digital camera with an AF-S Micro 105 mm 1:2.8 GED lens. Scanning electron microscopy (SEM) was performed with an FEI Quattro S at Yunnan University. Images were adjusted in Adobe Photoshop (brightness, contrast, colour balance, hue, saturation) and figures constructed in CorelDraw X7. All specimens, peels and slides are deposited in the Institute of Deep Time Terrestrial Ecology, Yunnan University and the Institute of Botany, Chinese Academy of Sciences, Beijing.

## RESULTS

### Systematic palaeobotany

Order: Noeggerathiales Nemejc 1931).

Family: Dorsalistachyaceae Wang *et al.* (2017).

Genus: *Paratingia*Zhang (1987) emend Qin, He et Wang.

Emended generic diagnosis: Once-pinnate compound leaf with a dorsi-ventral rachis. Pinnules dimorphic, anisophyllous, alternately arranged in four rows; two rows of large pinnules on lower (abaxial) side with wide angle to the rachis, oval, elliptical to lanceolate, with semi-amplexicaulous and plagiotropic base and entire or dentate lateral margins and apex; two rows of small pinnules on upper (adaxial) side with acute angle to the rachis. Pinnules vascularized by vertical row of bundles that rotate 90° at divergence from the rachis. Venation gently divergent, dichotomous. Rachis with secretory cavities/channels in cortex and continuous C-shaped or inverted Ω-shaped vascular bundle with protoxylem at lateral margins from which pinnule traces originate. Multiseriate, multicellular trichomes distributed on lower (abaxial) surface of pinnule and/or rachis.

Remarks: The generic diagnosis of Zhang (1987) is emended to incorporate new features, including rachis shape and anatomy.

Type species: *Paratingia datongensis*Zhang (1987).

Species: *Paratingia fuyuanensis* Qin, He et Wang sp. nov.

Specific diagnosis: Large pinnules broadly elliptical with rounded apices and blades spreading in the horizontal plane. Margins of large and small pinnules with minute teeth lacking veins. Apex of small pinnules dividing into two or three teeth, each with a single vein. Abaxial pinnule surface with ribs corresponding to position of individual veins. Pinnule vascular bundles accompanied by mass of transfusion cells with reticulate wall pitting. Rachis surface and abaxial surface of large and small pinnules densely covered with multiseriate, multicellular trichomes.

**Fig. 1.**
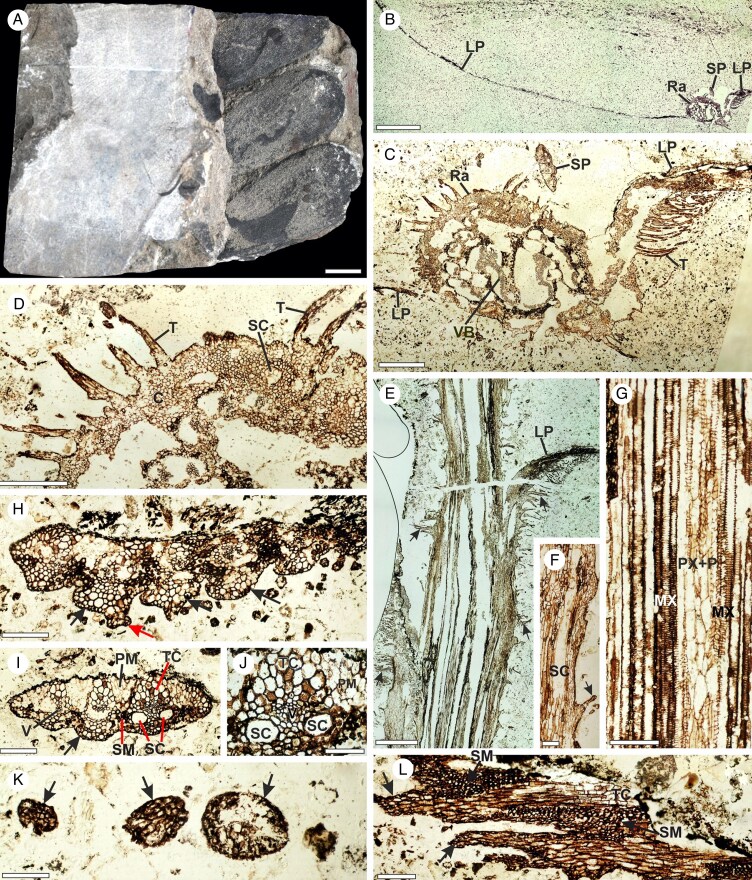
Vegetative leaves of *Paratingia fuyuanensis* sp. nov. Holotype (YNUPB11009). (A) Once-pinnate compound leaf with three large pinnules visible and other parts buried in the rock. Scale bar = 1 cm. (B) Leaf in cross-section showing gross morphology. Slide: YH0853. Scale bar = 5 mm. (C) Enlargement from (B) with elliptical rachis, inverted Ω-shaped vascular bundle and trichomes on large pinnule abaxial surface. Scale bar = 1 mm. (D) Enlargement from (C) showing trichomes on rachis surface, parenchymatous cortex and secretory canal. Scale bar = 0.5 mm. (E) Leaf in longitudinal section showing trichomes on rachis and large pinnule abaxial surface (arrows). Slide: YH0851. Scale bar = 2 mm. (F) Longitudinal section through rachis cortex showing secretory canal and trichome (arrow). Slide: YH0851. Scale bar = 100 μm. (G) Part of lateral margin of inverted Ω-shaped primary xylem strand in longitudinal section, showing mixed parenchyma cells and protoxylem tracheids surrounded by metaxylem tracheids with scalariform wall thickenings. Slide: YH0851. Scale bar = 200 μm. (H) Cross-section through part of a large pinnule with abaxial ribs (black arrows). Red arrow indicates a trichome on the rib. Slide: YH0826. Scale bar = 200 μm. (I) Cross-section through basal part of small pinnule showing abaxial ribs (arrow), veins comprising small tracheids, well-developed transfusion tissue above vein, parenchymatous and sclerenchymatous mesophyll, and two secretory canals located under vein. Slide: YH0853. Scale bar = 200 μm. (J) Enlargement from (I) showing vein, transfusion tissue and mesophyll. Scale bar = 100 μm. (K) Cross-section showing three teeth at the apex of small pinnule (arrows). Slide: YH0868. Scale bar = 100 μm. (L) Slightly oblique paradermal section of large pinnule with abaxial ribs (arrows) and sclerenchymatous mesophyll. Slide: YH0833. Scale bar = 200 μm. C, cortex; LP, large pinnule; MX, metaxylem; P, parenchyma; PM, parenchymatous mesophyll; PX, protoxylem; Ra, rachis; SC, secretory canal; SM, sclerenchymatous mesophyll; SP, small pinnule; T, trichomes; TC, transfusion cells; V, vein; VB, vascular bundle.

Holotype: YNUPB11009 (Fig. 1; Supplementary Data Figs S2 and S4–S6).

**Fig. 2.**
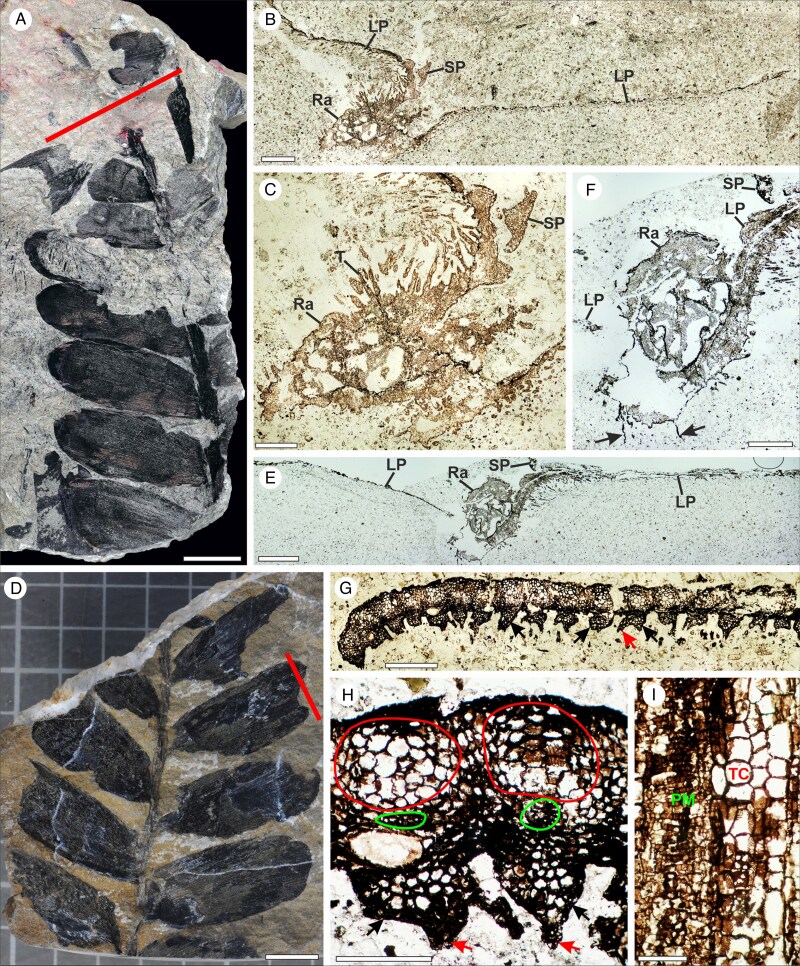
Vegetative leaves of *Paratingia fuyuanensis* sp. nov. (A–C) Specimen YNUPB11008. (A) Once-pinnate compound leaf with four large pinnules visible and the apex buried in the rock. Red line indicates the position of cross-section through the leaf. Scale bar = 2 cm. (B) Leaf in cross-section showing gross morphology. Slide: YH0882. Scale bar = 1 mm. (C) Enlargement from (2) showing rachis and a small pinnule. Scale bar = 0.5 mm. (D–I) Specimen 72014. (D) Hand specimen with red line indicating position of cross-section through a large pinnule. Scale bar = 1 cm. (E) Cross-section through leaf. Slide: WP2-0812. Scale bar = 2 mm. (F) Enlargement of a part of (E) showing rachis and a partially preserved small pinnule. Arrows indicate trichomes on abaxial rachis surface. Scale bar = 1 mm. (G) Cross-section of the large pinnule through the red line in (D) showing ribs (black arrows) on the abaxial side. Red arrow indicates a trichome on the rib. Slide: WP2-0829. Scale bar = 0.5 mm. (H) Enlargement of a part of (G) showing transfusion tissue (red circles), very small veins (green circles), secretory cavity and ribs (black arrows). Red arrows indicate trichomes on the rib. Scale bar = 200 μm. (I) Paradermal sections of the large pinnule showing transfusion cells and parenchymatous mesophyll. Slide: WP2-0817. Scale bar = 100 μm. Abbreviations as in Fig. 1 legend.

Other specimens: YNUPB11008 (Fig. 2A–C; Supplementary Data Fig. S1A–E); 72014 (Fig. 2D–I; Supplementary Data Figs S1F–H, S3 and S7).

Repository: YNUPB11009 and YNUPB11008 are deposited in the Institute of Deep Time Terrestrial Ecology, Yunnan University; 72014 is deposited in the Museum of the Institute of Botany, CAS, Beijing.

Type locality: Xuanwei, eastern Yunnan Province, China.

Geological unit: Xuanwei Formation.

Age: Lopingian (late Permian).

Etymology: The specific name refers to the type locality, Fuyuan County.

Species: *Paratingia qingyunensis* Qin, He et Wang sp. nov.

Diagnosis: Rachis glabrous. Large pinnules elliptical with sub-rounded apices and blades at an angle to bedding planes. Entire margins of large and small pinnules. Apex of small pinnules entire. Abaxial surface of large and small pinnules smooth. Stomatal apparatuses on abaxial surface of large pinnules randomly distributed, anomocytic, with long axes of stomatal apertures parallel to veins. Dense multiseriate, multicellular trichomes restricted to abaxial surface of only some pinnule bases.

**Fig. 3.**
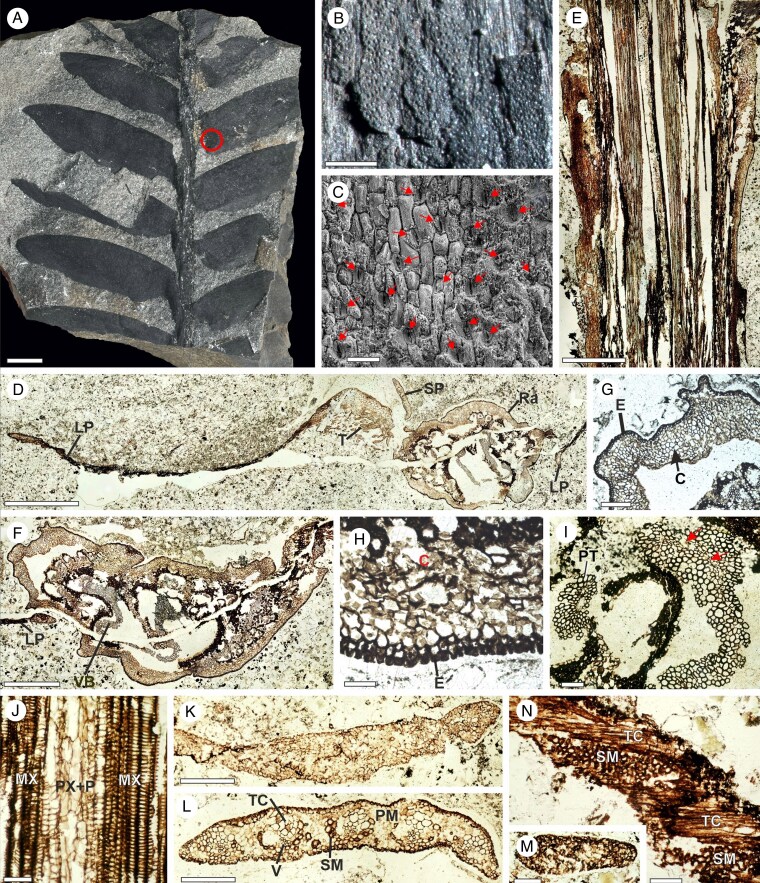
Vegetative leaves of *Paratingia qingyunensis* sp. nov. type specimen (YNUPB11007). (A) Once-pinnate compound leaf with area in red circle preserving epidermis. Scale bar = 1 cm. (B) Epidermis from red circle in (A) under stereo microscope showing randomly distributed stomatal apparatuses. Scale bar = 0.5 mm. (C) Epidermis under scanning electron microscope, showing stomatal apparatuses (red arrows). Scale bar = 50 μm. (D) Cross-section through leaf showing rachis with U-shaped vascular bundle, no trichomes on rachis surface, and a small pinnule above rachis. Slide: YH0788. Scale bar = 2 mm. (E) Longitudinal section through rachis showing no trichomes on surfaces. Slide: YH0769. Scale bar = 2 mm. (F) Cross-section through rachis showing inverted Ω-shaped vascular bundle and no trichomes. Slide: YH0759. Scale bar = 1 mm. (G, H) Cross-sections through part of the rachis showing parenchymatous cortex and epidermis (labelled E) consisting of cells with papillary outer periclinal walls. Slide: YH0759. Scale bar = 200, 50 μm. (I) Cross-section through part of the rachis showing lateral margin of inverted Ω-shaped xylem strand with two protoxylem poles (arrows) and a pinnule trace (PT). Slide: YH0761. Scale bar = 100 μm. (J) Longitudinal section through lateral margin of inverted Ω-shaped xylem strand showing metaxylem (MX) and area comprising parenchyma and protoxylem (PX + P). Slide: YH0769. Scale bar = 50 μm. (K) Cross-section through part of a poorly preserved large pinnule lacking ribs and trichomes on the abaxial surface. Slide: YH0772. Scale bar = 200 μm. (L) Enlargement of small pinnule from (D) with well-developed transfusion tissue; it lacks ribs and trichomes on abaxial surface. Randomly distributed cells of sclerenchymatous mesophyll are very different from the pinnules of *Paratingia fuyuanensis* sp. nov. (compare Fig. 1I with Fig. 3L). Scale bar = 200 μm. (M) Entire apex of a small pinnule in cross-section. Slide: YH0805. Scale bar = 100 μm. (N) Paradermal section of a large pinnule. Slide: YH0806. Scale bar = 100 μm. Abbreviations as in Fig. 1 legend.

**Fig. 4.**
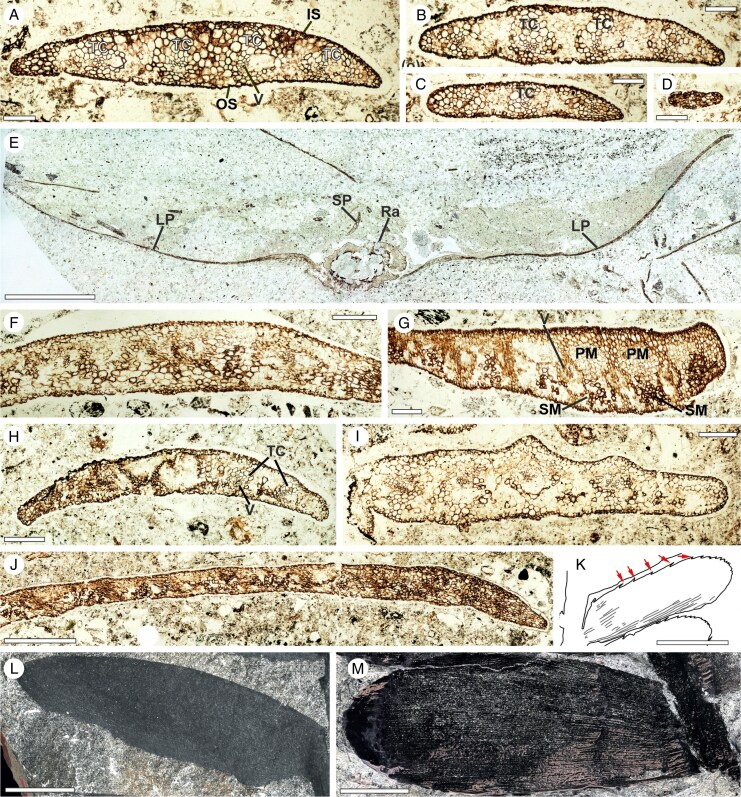
*Paratingia qingyunensis* sp. nov. (A–D) Type specimen (YNUPB11007). Acropetal successive cross-sections of a small pinnule from the mid-region in (A) to the entire apex in (D). Slides: HY0815, HY0815, HY0816, HY0816. Scale bar =100 μm. (E–J) Specimen YNUPB11010. (E) Cross-section through leaf. Scale bar = 5 mm. (F, G) Cross-sections of large pinnule showing the smooth abaxial side without trichomes. Slides: HY0886, HY0884. Scale bar = 200 μm. (H, I) Cross-sections of small pinnule showing the smooth outer-facing (= abaxial) side without trichomes. Slides: HY0886, HY0885. Scale bar = 200 μm. (J) Oblique cross-section of a large pinnule showing the smooth abaxial side without trichomes. Slide: HY0886. Scale bar = 0.5 mm. (K) A pinnule or leaflet of the holotype of *Plagiozamites oblongifolius* Halle with an oblong shape and distinct teeth (arrows) on the margin (redrawn from Halle, 1927: Pl. 60, Fig. 6 and in the Swedish Museum of Natural History). Scale bar = 1 cm. (L, M) A large pinnule of *Paratingia qingyunensis* sp. nov. and *P*. *fuyuanensis* sp. nov., respectively, without distinct teeth on the margin and with an elliptical shape and much larger size than that of the type specimen of *Plagiozamites oblongifolius* Halle. Scale bar = 1 cm. IS, inner-facing surface (= adaxial surface); OS, outer-facing surface (= abaxial surface); other abbreviations as in Fig. 1 legend.

Holotype: YNUPB11007 (Figs 3 and 4A–D; Supplementary Data Figs S8–S10).

Paratype: YNUPB11010 (Fig. 4E–J; Supplementary Data Figs S11–S13).

Depository: Institute of Deep Time Terrestrial Ecology, Yunnan University.

Etymology: The specific name refers to the type locality, Qingyun coal mine.

### Summary descriptions

The specimens each comprise the central portion of a once-pinnate compound leaf and share multiple features that show they belong to closely related species. Pinnules are of two sizes. Large pinnules are attached to the rachis laterally and their bases are plagiotropic or inclined forward, whereas small pinnules project above the rachis in some cross-sections of the leaf. When exposed on the sediment, only two rows of pinnules are visible, attached to the rachis alternately and obliquely (Figs 1A, 2A, D and 3A; Supplementary Data Fig. S1A, F). Individual pinnules are 15 mm or more wide, more than 35 mm long, with the margins and apices entire and no prominent teeth discernible (Fig. 4L, M; Supplementary Data Fig. S8A). Several veins extend from the pinnule base to the margins and apex and do not branch, with ∼30 veins per centimetre in the middle part of the pinnule.

Cross-sections of the leaf show that the leaf rachis is thin, 5–6 mm in diameter, and centrally has a large, continuous, and inverted Ω-shaped primary xylem strand (Figs 1C, 2F and 3F; Supplementary Data Figs S1H, S2A and S3B), here termed an Ω-strand. The Ω-strand is three to five tracheids thick with protoxylem positioned at the lateral margins (Fig. 3I; Supplementary Data Fig. S3B). Metaxylem tracheids possess scalariform thickenings on longitudinal cell walls (Figs 1G and 3J; Supplementary Data Fig. S2E). The Ω-strand is encircled by a sclerenchymatous sheath (Figs 1C, 2F and 3D, F; Supplementary Data Figs S1H, S2A, S3B and S12B). Two traces diverge from each lateral margin of the Ω-strand and extend toward the adaxial and abaxial side of the rachis, respectively, and each of them divide several times to form at least eight to ten small traces in total with the adaxial two or three vascularizing a small pinnule and the rest enter a large pinnule (Supplementary Data Fig. S4B–I). At the level where the pinnule diverges from the rachis it rotates 90° so that the row of bundles extends into the pinnule in the same plane as the rest of the frond.

Cortex of the rachis consists of parenchyma cells, which are isodiametric in cross-section (Figs 1D and 3G, H; Supplementary Data Figs S3A and S12C) and elongate in longitudinal section (Fig. 1F; Supplementary Data Figs S2C, D and S8D). Secretory canals are present in the cortex (Fig. 1D; Supplementary Data Fig. S2B, C).

Tracking leaf anatomy through successive cross-sections of the rachis demonstrates that the small pinnules originate from the upper (adaxial) surface of the rachis and extend alternately in two rows (Supplementary Data Figs S5 and S9). Individual small pinnules are oriented parallel to, and hence lie flat on, the rachis. Small pinnules are less than 10 mm long and up to 2 mm wide, with no more than six veins in the mid-region. Cross-sections of large and small pinnules reveal the veins have very small tracheids with diameters of 10–15 μm and a poorly developed bundle sheath (Figs 1H–J, 2H, 3L and 4A, B, F–I; Supplementary Data Figs S3D, S6B, G and S13F). Phloem is not preserved. A mass of transfusion cells connects the veins and the adaxial epidermis (Figs 1H–J, 2H, 3L and 4A–C, G, H; Supplementary Data Figs S3D, S6B–E, G, H, S7F, S10M–O and S13F). Mesophyll consists of parenchyma cells neighbouring the adaxial epidermis and sclerenchyma cells neighbouring the abaxial epidermis (Figs 1I, 3L and 4G; Supplementary Data Figs S6G and S7G). Sclerenchyma cells are small, 15–25 μm in diameter, spherical, with cell walls up to 7–8 μm thick. Pinnules bear multiseriate, multicellular trichomes (Fig. 1C, 2C, F and 3D; Supplementary Data Fig. S2F, G).

**Fig. 5.**
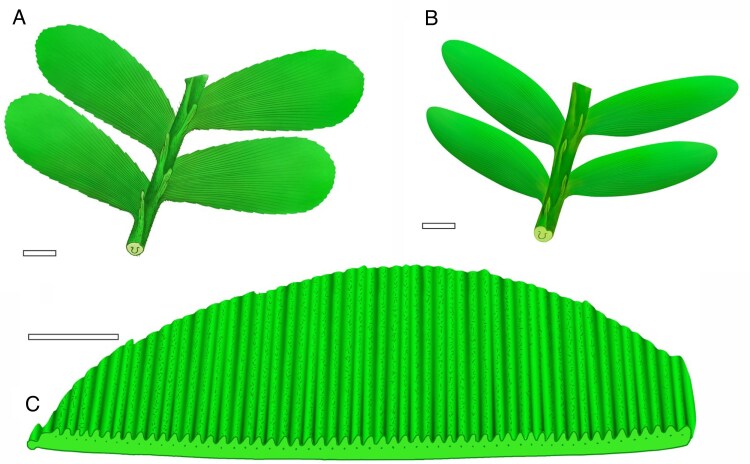
Suggested reconstruction of the two new species of *Paratingia.* (A) *Paratingia fuyuanensis* based on specimen YNUPB11009. Scale bar = 1 cm. (B) *Paratingia qingyunensis* based on specimen YNUPB11007. Scale bar = 1 cm. (C) Abaxial surface of the apical part of the large pinnule of *P*. *fuyuanensis* showing longitudinal ribs and trichomes. Scale bar = 0.2 mm.

**Fig. 6.**
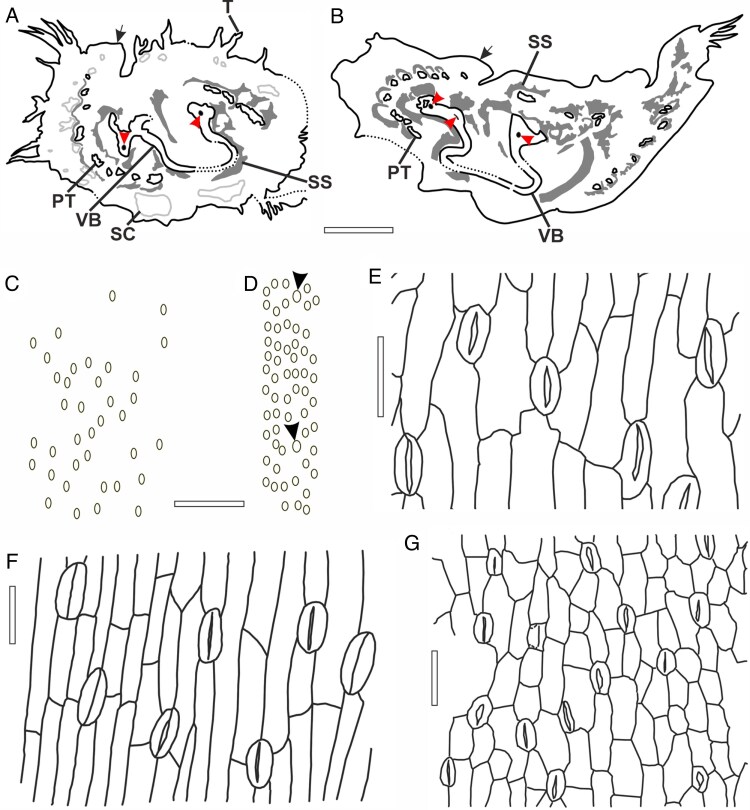
Line drawings emphasizing key features of leaves from the Xuanwei Formation. (A, B) Cross-sections through rachises of *Paratingia fuyuanensis* sp. nov. and *P*. *qingyunensis* sp. nov., respectively. Red arrowheads indicate protoxylem poles at lateral margins of inverted Ω-shaped vascular bundle; black arrows indicate small pinnule bases. Scale bar = 1 mm. (C, D) Distribution of stomatal apparatuses on the abaxial epidermis of *P. qingyunensis* sp. nov. and *Plagiozamites oblongifolius* Halle, respectively. (D) Tracing from image in Fig. 2E of Feng *et al.* (2017), with arrowheads indicating two comparatively large stomatal apparatuses. Scale bar = 200 μm. (E–G) Comparison of cellular structure from the lower (abaxial) epidermis of selected Noeggerathiales leaves. (E) *Noeggerathia foliosa* Sternberg from Šimůnek and Bek (2003). Scale bar = 50 μm. (F) *Tingia carbonica* Schenk from Liu *et al.* (1998). Scale bar = 50 μm. (G) *Paratingia qingyunensis* sp. nov. from this paper. Scale bar = 50 μm. PT, pinnule trace; SC, secretory canal; SS, sclerenchymatous sheath; VB, inverted Ω-shaped vascular bundle.

Despite all specimens displaying the features described above, several significant differences discriminate two separate species. *Paratingia fuyuanensis* sp. nov. has large pinnules that are broadly elliptical with rounded apices (Figs 1A, 2A, D, 4M and 5A; Supplementary Data Fig. S1A, F), and the surface of the rachis is covered with thick, multiseriate, multicellular trichomes (Figs 1C–F, 2C, F, 5A and 6A; Supplementary Data Figs S1C, H, S2A, D, S3A and S4). This species is based on three specimens (YNUPB11008, YNUPB11009 and 72014) that comprise basally and apically incomplete once-pinnate compound leaves. Externally the compound leaf shows the rachis and large pinnules, but the small pinnules are not visible due to incomplete preservation. In *P. fuyuanensis* the abaxial surface of both large and small pinnules possesses ribs that correspond directly to the positions of the veins (Figs 1H, I, 2G, H and 5C; Supplementary Data Figs S2F, S3C, D and S6B, C). Transfusion cells are well developed in both large and small pinnules (Figs 1I, J and 2H; Supplementary Data Figs S2F, S3D, S6C–E, G, H and S7B–F), and the lower (abaxial) pinnule surface is densely covered by thick, multiseriate, multicellular trichomes (Fig. 5C; Supplementary Data Fig. S2G). Apices of small pinnules are toothed and each tooth possesses a vein (Fig. 1K; Supplementary Data Figs S5I, O and S6F).

In *Paratingia qingyunensis* sp. nov., large pinnules are elongate elliptical with sub-rounded apices (Figs 3A, 4L and 5B; Supplementary Data Fig. S8A), and the rachis surface is smooth and lacks trichomes (Figs 3D, F and 6B; Supplementary Data Figs S9, S11A, B and S12B). The abaxial surface of large and small pinnules is flat and lacks ribs (Figs 3K, L and 4F–J; Supplementary Data Figs S8G, S10F–P, S11B, S12A and S13), and transfusion tissue is poorly developed in large pinnules (Figs 3K and 4F, G, J; Supplementary Data Fig. S13A, B, D). Thick, multiseriate, multicellular trichomes are present only on the abaxial surface of the basal parts of some large pinnules (Fig. 3D; Supplementary Data Fig. S9D–G), and the apices of small pinnules are entire and lack teeth (Figs 3M and 4D; Supplementary Data Figs S9O, S10L, P, S12A, B and S13C, E).

One specimen of *Paratingia qingyunensis* preserves a small area of epidermis on the abaxial surface of a large pinnule (Fig. 3A, B). This was investigated by stereoscopic and scanning electron microscopy. The epidermis lacks distinct costal and intercostal zones. Stomatal apparatuses are distributed randomly and do not form rows (Fig. 3B, C; Supplementary Data Fig. S8B) and consist of two slightly sunken guard cells surrounded by ordinary epidermal cells. Subsidiary cells are absent. Stomatal apparatuses are absent on the trichomes.

Full detailed descriptions of the two species are provided in the online Supplementary Materials.

## DISCUSSION

Successive cross-sections of the rachis of the two new species of *Paratingia* described and named in this paper support our knowledge that leaves of noeggatherialean progymnosperms bear four rows of pinnules that comprise two rows of large pinnules and two rows of small pinnules. In the Permian, this arrangement is characteristic of the Noeggerathiales genera *Tingia* Halle and *Paratingia* Zhang (e.g. Wang *et al.*, 2021*a*, *b*). Pinnules of *Tingia* usually have parallel venation and truncated and deeply lobed or dentate apices, whereas *Paratingia* pinnules have gently divergent venation and acute apices. The specimens we document have pinnules with gently divergent venation and apices that are not divided or truncated; we therefore assign them to *Paratingia*.

Three species of *Paratingia* have been reported previously: *P. datongensis* Zhang (Zhang, 1987; Sun *et al.*, 1999), *P. wudensis* Wang (Wang *et al.*, 2009) and *P. wuhaia* Wang *et al.* (Wang *et al.*, 2021*a*). In these species, the large pinnules have tapering apices with distinct teeth, but the large pinnules in both species from the Xuanwei Formation have rounded to sub-rounded apices and lack teeth. Small pinnules in the species from the Xuanwei Formation are <10 mm long and <2 mm wide, whereas they are much larger in the previously recognized species: 20–30 mm long and 6–8 mm wide in *P. datongensis* (Zhang, 1987; Sun *et al.*, 1999), up to 25 mm long and 2–2.5 mm wide in *P. wudensis* (Wang *et al.*, 2009) and up to 17 mm long and 9 mm wide in *P. wuhaia* (Wang *et al.*, 2021*a*). Collectively these features demonstrate that the specimens from the Xuanwei Formation are distinct and include two new species that we name *Paratingia fuyuanensis* Qin, He et Wang (specimens YNUPB11008, YNUPB11009 and 72014) and *Paratingia qingyunensis* Qin, He et Wang (specimens YNUPB11007 and YNUPB11010).

From the Xuanwei Formation, Guo *et al.* (1990) documented the morphology and anatomy of a leaf that they assigned to *Plagiozamites oblongifolius* Halle. In their specimen, the rachis has a continuous, inverted Ω-shaped vascular bundle with two outcurved (excurved) lateral margins in the centre; metaxylem tracheid walls possess scalariform thickenings and the vascular bundle is enclosed by a dark-coloured sclerenchymatous sheath that Guo *et al.* misinterpreted as phloem. The specimen also has prominent secretory channels in the rachis cortex and pinnule mesophyll, and there are transfusion cells in the mesophyll. These features are consistent with those of the specimens that we describe from the Xuanwei Formation. Guo *et al.* (1990) did not document rows of small pinnules arising from the adaxial side of the rachis. We suspect that there are two reasons that they did not recognize small pinnules. One is that in the rachis cross-section figured in their Plate I, 5 (Guo *et al.*, 1990), a small pinnule may be present in the section through the middle or apical parts of the small pinnule at a level where the small pinnule is not directly connected to the rachis. In this case, the small pinnules might be ignored or mistaken for leaves of another kind of plant. The other is that, in our study, not every cross-section through the rachis passed through a small pinnule due to the spaces between neighbouring small pinnules; it is likely that the cross-section illustrated by Guo *et al.* was positioned between small pinnules. Additional cross-sections of the rachis in their specimen are needed to verify this supposition.

We conclude that the specimens illustrated by Guo *et al.* (1990) also have small pinnules and conform to *Paratingia* rather than *Plagiozamites oblongifolius*. However, there are some differences between specimens investigated by Guo *et al.* and those that we document. From the illustrations of Guo *et al.* (1990), cortical cells of the rachis appear thick-walled and smaller than the metaxylem tracheids, and the inverted Ω-shaped xylem strand has smaller tracheids distributed abaxially that caused them to misinterpret the xylem strand as exarch. In both *Paratingia fuyuanensis* sp. nov. and *P. qingyunensis* sp. nov., cortical cells of the rachis are thin-walled and slightly larger than or as large as the metaxylem tracheids, and tracheids at the abaxial and adaxial sides of Ω-shaped bundle are the same size. Specimens investigated by Guo *et al.* lack densely distributed trichomes on the rachis, which is very different from *P. fuyuanensis*. We conclude that specimens investigated by Guo *et al.* most likely represent a third species of *Paratingia* from the Xuanwei Formation. We do not formally name and diagnose this third species of *Paratingia* from the Xuanwei Formation as it requires additional specimens and information to more comprehensively characterize it.

From the same locality as our specimens in the Xuanwei Formation, Feng *et al.* (2017) documented epidermal features from cuticles which they interpreted to belong to *Plagiozamites oblongifolius*. They showed three partially preserved, once-pinnate compound leaves (their Fig. 1A–C, respectively). Pinnules (= leaflets of Feng *et al.*, 2017) shown in Fig. 1A and C of Feng *et al.* (2017) are broadly ovate and somewhat resemble our specimens of *Paratingia fuyuanensis* sp. nov. However, the pinnules illustrated in their Fig. 1B are significantly different, being narrow and oblong. This morphology is closer to the type specimens of *Plagiozamites oblongifolius* (Halle, 1927) from lower Permian strata of North China, but specimens from the Xuanwei Formation are typically ∼70 mm long and 20 mm wide. This is much larger than the type specimens, which are typically <40 mm long and <10 mm wide (comparing Fig. 4K with Fig. 4L, M in this paper). Feng *et al.* (2017: Fig. 2A–C) illustrated three pieces of cuticle with teeth on the pinnule lateral margins and apices. The lower (abaxial) epidermis comprises well-defined costal and intercostal zones that are <200 μm wide; stomatal apparatuses are distributed in the intercostal zone and arranged roughly in longitudinal rows. Sporadic larger stomatal apparatuses occur irregularly among the smaller examples (Fig. 6D in this paper). Smaller stomatal apparatuses have four to six subsidiary cells and vary slightly in shape and size. The interior walls of the subsidiary cells bounding the stomatal aperture are much more strongly cutinized than the exterior walls, commonly forming a prominent ring-like structure circling the stomatal aperture. However, our specimens of *Paratingia* lack well-defined costal and intercostal zones on the abaxial epidermis, and stomatal apparatuses are distributed in a rather wider region that exceeds 500 μm; moreover, they are arranged irregularly, with apertures oriented in the same direction (Fig. 6C in this paper). Furthermore, in the new *Paratinia* specimens, stomatal apparatuses are approximately the same size and shape; they lack a prominent ring-like cutinized structure circling the stomatal aperture. Despite superficial similarities in leaf morphology and coming from the same location and formation, our specimens are clearly taxonomically distinct from those illustrated by Feng *et al.* (2017).

Regarding the affinity of *Plagiozamites oblongifolius*, both Guo *et al.* (1990) and Feng *et al.* (2017) concluded that their specimens belonged within Cycadales. Guo *et al.* (1990) based on their comparison of the inverted Ω-shaped vascular bundle in the rachis of *P. oblongifolius* with extant *Cycas* L., noting their overall similarity. However, the rachis of *Cycas* and other cycads possesses several small and individual vascular bundles, which are arranged in an inverted Ω-shape, each possessing its own protoxylem strand (Matte, 1904; Stevenson, 1992; Hermsen *et al.*, 2007). By contrast, the rachis of our specimens and those illustrated by Guo *et al.* (1990) has a single, long, and continuous inverted Ω-shaped vascular bundle with protoxylem located at the lateral margins. These features are clearly distinct from those of *Cycas* but resemble the rachis anatomy of *Paratingia wuhaia* (Wang *et al.*, 2021*a*) and *Tingia unita* (Wang *et al.*, 2021*b*). Feng *et al.* (2017) based their systematic conclusions on the presence of diagnostic cuticle features comprising deeply sunken haplocheilic stomatal apparatuses and strongly cutinized ring-like structures circling the stomatal aperture, features that also occur in extant Cycadales, such as *Macrozamia* Miquel and *Encephalartos* Lehmann. Unfortunately, Feng *et al.* (2017) did not indicate which of the specimens shown in their Figs 1A–C the illustrated cuticles were isolated from, complicating full characterization because they illustrated two species of pinnule (see above). Of these, the specimen in their Fig. 1B has oblong pinnules and is very similar to the type specimens of *Plagiozamites oblongifolius* from the early Permian (Halle, 1927) but differs from the specimens of *Paratingia* we document, which have elliptical pinnules. Thus, leaves previously assigned to *Plagiozamites oblongifolius* from the late Permian of south-western China actually represent more than one higher taxon. Some, such as *Paratingia fuyuanensis* sp. nov. and *Paratingia qingyunensis* sp. nov., are Noeggerathiales, whereas others, such as *Plagiozamites oblongifolius*, are Cycadales. These characters reveal hidden or cryptic diversity in fossil plant assemblages preserved as compressions/impressions in which diagnostic anatomical features required to distinguish different taxa are not preserved. From our study, this hidden diversity is extremely significant, as it encompasses *at least* five plant species from two distinct orders. In future studies where *Plagiozamites oblongifolius*-like once-pinnate leaves are encountered, special attention to the number of rows of pinnules that are present will be required to accurately discriminate between these otherwise cryptically distinct taxa. Detailed examination will be required to identify the small adaxial pinnules overlying the rachis typical of species of *Paratingia*. However, care must be taken to focus such examinations on the adaxial surface of fronds, as the smaller pinnules are not visible in abaxial views. This may not be possible in specimens preserved by coalified compression or impression. We have examined photographs of the type specimens of *Plagiozamites oblongifolius* Halle (Halle, 1927), as well as additional specimens of the species from the type locality at Nanjing Institute of Geology and Palaeontology, and did not find any evidence of small pinnules, confirming previous observations (Halle, 1927).

The epidermal structure of *Paratingia qingyunensis* sp. nov. is simple and consists of epidermal cells and stomatal apparatuses without specialized subsidiary cells; thus, the stomatal apparatuses are anomocytic (Fig. 6G in this paper). Previously recognized Noeggerathiales are also anomocytic and lack subsidiary cells, including *Tingia carbonica* (Schenk) Halle (Liu *et al.*, 1998), *Conchophyllum suboblongifolius* Cleal et Wang (Cleal and Wang, 2002), *Noeggerathia foliosa* Sternberg (Šimůnek and Bek, 2003) and three types of mesofossils with presumed Noeggerathiales affinity (Chen *et al.*, 2021) (Fig. 6E, F in this paper). Thus, the epidermal structure of the present specimens offers further evidence that they are of noeggerathialean affinities.

The specimens we document represent the first record of the typical noeggerathialean vegetative leaf *Paratingia* from the upper Permian strata of south-western China. From the Xuanwei Formation, two kinds of Noeggerathiales progymnosperm fertile organs have been documented previously. These are *Discinites* cf. *orientalis* Gu et Zhi (Zhao *et al.*, 1980) and *Dorsalistachya quadrisegmentorum*Wang *et al.* (2017). This confirms the existence of *at least* two noeggerathialean whole-plant species within the formation.

Leaves of *Paratingia fuyuanensis* and *P. qingyunensis* share important anatomical features with *Dorsalistachya quadrisegmentorum*, which co-occurs in the Xuanwei Formation. These include an inverted Ω-shaped vascular bundle with protoxylem located in the lateral margins, ‘sporophyll’ and pinnule traces arising from the lateral margins of the Ω-shaped bundle, and prominent secretory channels in the cortex of the pseudostrobilar axis/leaf rachis and ‘sporophyll’/pinnule mesophyll (Wang *et al.*, 2017). The shared features demonstrate that these fossils constitute closely related species within Noeggerathiales family Dorsalistachyaceae Wang *et al.* (Wang *et al.*, 2017). However, when Wang *et al.* (2017) established Dorsalistachyaceae, *Paratingia* was unknown in the Xuanwei Formation, and they interpreted *Plagiozamites oblongifolius* to be the vegetative leaf of the family. It is now clear that *Paratingia* should be regarded as the vegetative leaves of Dorsalistachyaceae (Wang *et al.*, 2017), whereas *Plagiozamites oblongifolius* should be excluded from Noeggerathiales and retained within Cycadales. Thus, our results clearly demonstrate that a taxon initially established as a single fossil species (= ‘organ-species’ of Bateman and Hilton, 2009) can actually represent two taxa widely accepted as separate orders and *at least* five distinct species, graphically illustrating the challenges posed by palaeobotanical taxonomy for understanding past floral composition and diversity (Bateman and Hilton, 2009). Despite superficial resemblance, it is now clear that isolated leaves of Noeggerathiales and cycads can be distinguished through a combination of their gross morphology, rachis anatomy and epidermal characters (Table 1), but detailed investigations are required to achieve that end. As we elaborate above, this may not be possible in specimens with compression/impression preservation, especially where the adaxial leaf surface is embedded in the matrix. Such instances may necessitate physical preparation of the specimen to check the adaxial morphology to search for small pinnules (e.g. Mamay, 1968; Wang *et al.*, 2009).

**Table 1.** Comparison of key features in Noeggerathiales and Cycadales leaves.

Foliar character	Noeggerathiales	Cycadales
Foliar morphology	Monopinnate	Monopinnate and bipinnate
Pinnule organization on rachis	Two rows and four rows	Two rows
Rachis vascularization	Inverted Ω-shaped vascular strand comprising a single long and continuous bundle with the protoxylem bundles located at lateral margins	Inverted Ω-shaped vascular strand comprising many small individual bundles each with its own protoxylem bundle
Mucilage canals/cavities	In rachis and pinnules	In rachis and pinnules
Pinnule midrib	Absent	Present
Pinnule venation	Parallel and radiate	Parallel and radiate
Trichome morphology	Multicellular and multiseriate	Filiform or hair-like, consisting of one or two cells
Stomatal apparatuses	Anomocytic, with guard cells and no subsidiary cells	Haplocheilic, with guard and subsidiary cells

Several apparent morphological similarities between Noeggerathiales and Cycadales demand closer attention, particularly in terms of distinguishing plesiomorphic features from those deemed parallel or convergent. As reviewed by Scotland (2011), several ways of distinguishing convergence from parallelism have been proposed. For some observers, parallel states emerge from close relatives, but convergent states derive from more distant relatives. For others, convergent features should have contrasting ancestral states, and for yet others convergent features should be underpinned by contrasting developmental genetics. Scotland (2011) eventually concluded that for any such evolutionary step, the morphological transition is convergent, but the underlying genetic change is by definition parallel, since no two independent genetic changes will in practice be identical.

Addressing fossil phenotypes precludes exploration of their underlying genetics, but relatedness of taxa and inferences of ancestral states can be made. As heterosporous progymnosperms, Noeggerathiales were not closely related to Cycadales gymnosperms (Wang *et al.*, 2021*a*), even if in some instances they co-inhabited the same growth environments (e.g. Pfefferkorn and Wang, 2016). Progymnosperms represent the evolutionary stem group from which seed plants arose (e.g. Rothwell and Serbet, 1994; Hilton and Bateman, 2006), such that morphological and anatomical similarities between Noeggerathiales and cycads could reflect either shared ancestry or convergent evolution. Noeggerathiales and cycads have upright trunks with leaf bases and an apical crown of pinnate leaves, but this differs from the architecture of the earliest seed plants evolutionarily positioned between them, including *Elkinsia* and *Heterangium*, which suggests convergence in growth forms between Noeggerathiales and cycads. This is further supported by differences in wood type, which is pycnoxylic in Noeggerathiales (Wang *et al.*, 2021*b*) and manoxylic in cycads (e.g. Rothwell and Serbet, 1994; Taylor *et al.*, 2009), suggesting parallel evolution of growth habits. Both Noeggerathiales and Cycadales have pinnate leaves (Table 1) with parallel venation, but this is a shared plesiomorphic condition in lignophytes (progymnosperms plus seed plants) (e.g. Rothwell and Serbet, 1994; Rothwell, 1999) so likely reflects shared ancestry.

A potential relationship between Noeggerathiales and Cordaitales also needs to be evaluated. Characters such as parallel venation, sclerenchymatous cell nests, abaxial grooves and ridges, and abaxial trichomes observed in *Paratingia* leaves also occur in leaves of *Noeggerathiopsis* and *Rufloria* (Cordaitales) from the Permian Gondwanan flora (e.g. McLoughlin and Drinnan, 1996; Taylor *et al.*, 2009). Abaxial grooves and ridges and abaxial trichomes may be related to environmental conditions where the plant grew, representing convergent evolution between Cordaitales and Noeggerathiales in response to growing in mire settings.

As a final point, we highlight that prior to the assignment here of leaves of *Paratingia* to the family Dorsalistachyaceae, this kind of leaf was previously also assigned to the noeggerathialean family Tingiostachyaceae (Wang *et al.*, 2017), borne on the whole-plant *Paratingia wuhaia* (Wang *et al.*, 2021b). This co-occurrence suggests the Tingiostachyaceae and Dorsalistachyaceae are closely related and distinguished on other parts of the whole-plant morphology, in this case their fertile structures (Wang *et al.*, 2017). This situation is not unique to the Noeggerathiales; in the lepidodendralean arborescent lycopsids the leaf genus *Lepidophylloides* Snigirevskya is shared by the families Lepidodendraceae and Diaphrodendraceae (Bateman *et al.*, 1992). The same situation is also observed in Ginkgoales, conifers and Glossopteridales (Taylor *et al.*, 2009). These examples demonstrate evolutionary conservatism in vegetative organs compared with their more distinctive and complex fertile organs.

### Summary and conclusions

Documentation here of two new species of *Paratingia* and reinterpretation of leaves figured by Guo *et al.* (1990) as a third species extends the geographical and stratigraphical ranges of the genus that were previously restricted to the species *Paratingia datongensis*, *P. wudensis* and *P. wuhaia* from the early Permian of the North China Plate into the late Permian of the South China Plate (Zhang, 1987; Sun *et al.*, 1999; Wang *et al.*, 2009, *2*021*a*). *Paratingia* can now be recognized as a distinctive and endemic Noeggerathiales genus from the late Palaeozoic Cathaysian (*Gigantopteris*) floras of the North and South China Plates, and as a member of the family Dorsalistachyaceae. In terms of species richness, we show that the plants previously recognized as *Plagiozamites oblongifolius* from the Xuanwei Formation comprise at least five distinct species, including three species of *Paratingia* and two potentially new species of *Plagiozamites*, both of which are distinct from *P. oblongifolius* from the early–middle Permian of North China. This recognition increases the diversity reported from the Xuanwei Formation (Dal Corso *et al.*, 2022; Xu *et al.*, 2022); simultaneously increasing the number of species to have fallen victim to the Permian–Triassic mass extinction and further elevating its perceived magnitude (e.g. Feng *et al.*, 2020; Dal Corso *et al.*, 2022; Xu *et al.*, 2022). This biotic crisis witnessed the extinction of the low-latitude, wetland, Cathaysian *Gigantopteris* rainforest ecosystem that included the loss of the species *Plagiozamites oblongifolius* (Xu *et al.*, 2022), which can now be recognized as the loss of three species of *Paratingia* and at least two species of *Plagiozamites.*

Present evidence suggests that the Cathaysian (*Gigantopteris*) flora, including *Paratingia*, migrated southwards from the North China Plate from the early and middle Permian into the late Permian of the South China Plate, presumably in response to climatic change and increasing aridity in North China through the Permian (Hilton and Cleal, 2007). The Cathaysian flora was in decline in the South China Plate prior to the Permian–Triassic mass extinction (Feng *et al.*, 2020; Dal Corso *et al.*, 2022; Xu *et al.*, 2022), but most of its floristic diversity, including Noeggerathiales, was lost during the extinction event itself. Exceptions to this pattern include the rare occurrences of holdover taxa, including *Gigantopteris* and *Gigantonoclea*, into the basal few metres of the Triassic Kayitou Formation (Xu *et al.*, 2022).

As documented here, both the noeggerathialean *Paratingia* and the cycad *Plagiozamites* in the Xuanwei Formation have superficially similar once-pinnate megaphyllous leaves. This undoubtedly has contributed to their previous conflation, thus masking the fact that only cycads survived the Permian–Triassic mass extinction event. This observation suggests that gross leaf morphology was not an important factor in determining which plant species lived and which died. Survivability was more likely related to their heterosporous reproductive biology, resilience to high temperature, and water usage, as climates changed from warm and wet to potentially lethally hot and dry (Dal Corso *et al.*, 2022; Xu *et al.*, 2022). The complete extinction of the Cathaysian (*Gigantopteris*) flora occurred slightly higher within the Kayitou Formation, but by that time the entire Noeggerathiales lineage, along with the lepidodendralean lycophytes and other wetland plant species, had already been consigned to the fossil record (Feng *et al.*, 2020; Xu *et al.*, 2022).

## Supplementary Material

mcaf272_Supplementary_Data

## SUPPLEMENTARY DATA

Supplementary data are available at *Annals of Botany* online and consist of the following. Appendix S1: Full description of species. Additional supporting information may be found online in the supporting information section at the end of the article. S1: detailed description of new species.
