# Supplementary material for: Convergent evolution in the late Permian megaphyllous leaves of the Noeggerathiales progymnosperm Paratingia and the cycad Plagiozamites
Source: Ann Bot. 2025 Oct 29;137(6):1745–57. doi: 10.1093/aob/mcaf272 (PMC13275014; doi:10.1093/aob/mcaf272)
Supplement: mcaf272_Supplementary_Data [file mcaf272_supplementary_data.docx]

**Convergent evolution in the late Permian megaphyllous leaves of the Noeggerathiales progymnosperm *Paratingia* and the cycad *Plagiozamites***

**Yi-Fei Qin^1^, Xiao-Yuan He^1,2,^*, Shi-Jun Wang^3^, Xinshi Cheng^4^, Jason Hilton^4,5^ and Gar W. Rothwell^6,7^**

*^1^Institute of Palaeontology, Yunnan Key Laboratory for Palaeobiology, Yunnan University, Kunming 650500, China, ^2^State Key Laboratory of Palaeobiology and Stratigraphy, Nanjing Institute of Geology and Palaeontology, Chinese Academy of Sciences, Nanjing 210008, China, ^3^State Key Laboratory of Systematic and Evolutionary Botany, Institute of Botany, Chinese Academy of Sciences, Beijing 100093, China, ^4^School of Geography, Earth and Environmental Sciences, University of Birmingham, Edgbaston, Birmingham, B15 2TT, UK, ^5^Birmingham Institute of Forest Research, University of Birmingham, Edgbaston, Birmingham, B15 2TT, UK, ^6^Department of Environmental and Plant Biology, Porter Hall, Ohio University, Athens, Ohio, 45701 USA, ^7^Department of Botany and Plant Pathology, Cordley Hall, Oregon State University, Corvallis, Oregon, 97331, USA*

* *For correspondences. Email* [*hexiaoy@ynu.edu.cn*](mailto:hexiaoy@ynu.edu.cn), j.m.hilton@bham.ac.uk

**Full description of specimens**

**Species** *Paratingia fuyuanensis* Qin, He *et* Wang sp. nov.

**Morphological features**

The new species is based on three specimens (YNUPB11008, YNUPB11009 and 72014) that comprise basally and apically incomplete once pinnate compound leaves. Externally, the compound leaf preserves the rachis and large pinnules, but the small pinnules are not visible due to incomplete preservation. Specimen YNUPB11009 is ca. 6 cm long and has one row of large pinnules exposed on the surface of the rock with the rachis and another row of large pinnules buried in the matrix (Fig. 1A). Specimen YNUPB11008 is ca. 10 cm long and its lower part is exposed on the surface of the rock whereas its upper part is buried in the matrix (Fig. 2A; Fig. S1A). The rachis and large pinnules of specimen 72014 are almost all present on the surface of the rock. The specimen is ca. 7 cm long, with only the rachis and some pinnule apices buried in the matrix (Fig. 2D; Fig. S1F). The rachis is thin and ca. 5 mm wide in YNUPB11008 and 2–3 mm wide in 72014. Large pinnules are arranged obliquely and alternately on the rachis at angles of 70°–80° from the rachis. Adjacent pinnules in the same row either contact each other or are separated by a small distance. Large pinnules are broadly elliptical with rounded apices and broad semi-amplexicaul bases. In specimens YNUPB11009 and YNUPB11008, large pinnules are ca. 6 cm long and 2.5 cm wide, whereas in specimen 72014 they are ca. 3.5 cm long and 1.5 cm wide. Length:width ratio of large pinnules in the three specimens are ca. 2.3–2.5:1. Lamina of the large pinnules spread out in a horizontal plane. Large pinnule margins and apices are entire and appear to lack teeth. Several veins extend from the pinnule base to the margins and apex. Veins are distinct with ca. 30 veins per centimeter in the middle of large pinnules.

**Anatomical features**

Rachis

In specimen YNUPB11009, the cross-sectional shape of the rachis usually is planoconvex with its upper (adaxial) surface roughly flat and the lower (abaxial) surface convex (Fig. S2A). However, in some cross-sections the rachis is somewhat elliptical (Fig. 1C). In YNUPB11008 and 72014, the rachis is mainly vertically elliptical in cross-section (Fig. 2C, F; Fig. S1C, H). The rachis is 5 mm wide and 3 mm high in YNUPB11009, 2.2–2.5 mm wide and 3.7 mm high in 72014, but only 1.5 mm wide and 2.5 mm high in the upper part of specimen YNUPB11008.

The rachis surface is covered with multiseriate and multicellular trichomes (Fig. 1C–F; Fig. 2C, F; Fig. S1C, D, H; Fig. S2A, D; Fig. S3A). In YNUPB11009, trichomes are better developed than in the other specimens. Trichomes vary greatly in length. Short trichomes have rounded or sharp tips and consist of isodiametric parenchyma cells (Fig. S3A). Long trichomes are up to 0.6 mm long, rod-like or spine-like, and consist of longitudinally rectangular cells with lengths of 30–100 μm or more (Fig. 1D; Fig. S1D). In cross-section trichomes are mostly circular with diameters of 50–100 μm, and consist of up to 10 or more cells with diameters of 20–30 μm.

The epidermis is a single cell thick and comprises nearly isodiametric cells in cross-section with diameters of 10–18 μm. Epidermal cells possess thicker walls than those of cortical cells and their outer periclinal wall is thicker than other walls.

The cortex varies in thickness from several to more than ten layers of cells, comprising parenchyma cells which are nearly isodiametric and polygonal in cross-section with diameters of 30–35 μm (Fig. 1D; Fig. S3A). In longitudinal section, several layers of cells under the epidermis are slightly longitudinally elongate and polygonal, whereas those next to them are longitudinally elongate or rectangular up to 700 μm long (Fig. 1F; Fig. S2D).

In specimens YNUPB11009 and 72014, secretory canals are present in the cortex of the rachis. In YNUPB11008, no secretory canals have been found in the cortex, probably due to having only a single cross-section of the rachis. Secretory canals in the rachis are nearly circular or elliptical in cross-section, elongate in longitudinal section, and vary greatly in size. Secretory canals are empty or filled with large, isodiametric, thin-walled cells with diameters up to 60–70 μm (Fig. 1D, F; Fig. S2B, C).

In the center of the rachis, a large vascular bundle is surrounded by poorly preserved fundamental tissue. The large vascular bundle consists of a single long and continuous primary xylem strand without phloem preserved. In cross-section, the xylem strand is usually inverted Ω-shaped with two outcurved (excurved) lateral margins (Fig. 1C; Fig. 2F; Fig. S1H; Fig. S2A; Fig. S3B). However, in some cross-sections of the rachis, the xylem strand is more or less U-shaped (Fig. S1C). For the convenience of the description, we divide the inverted Ω-shaped xylem strand into


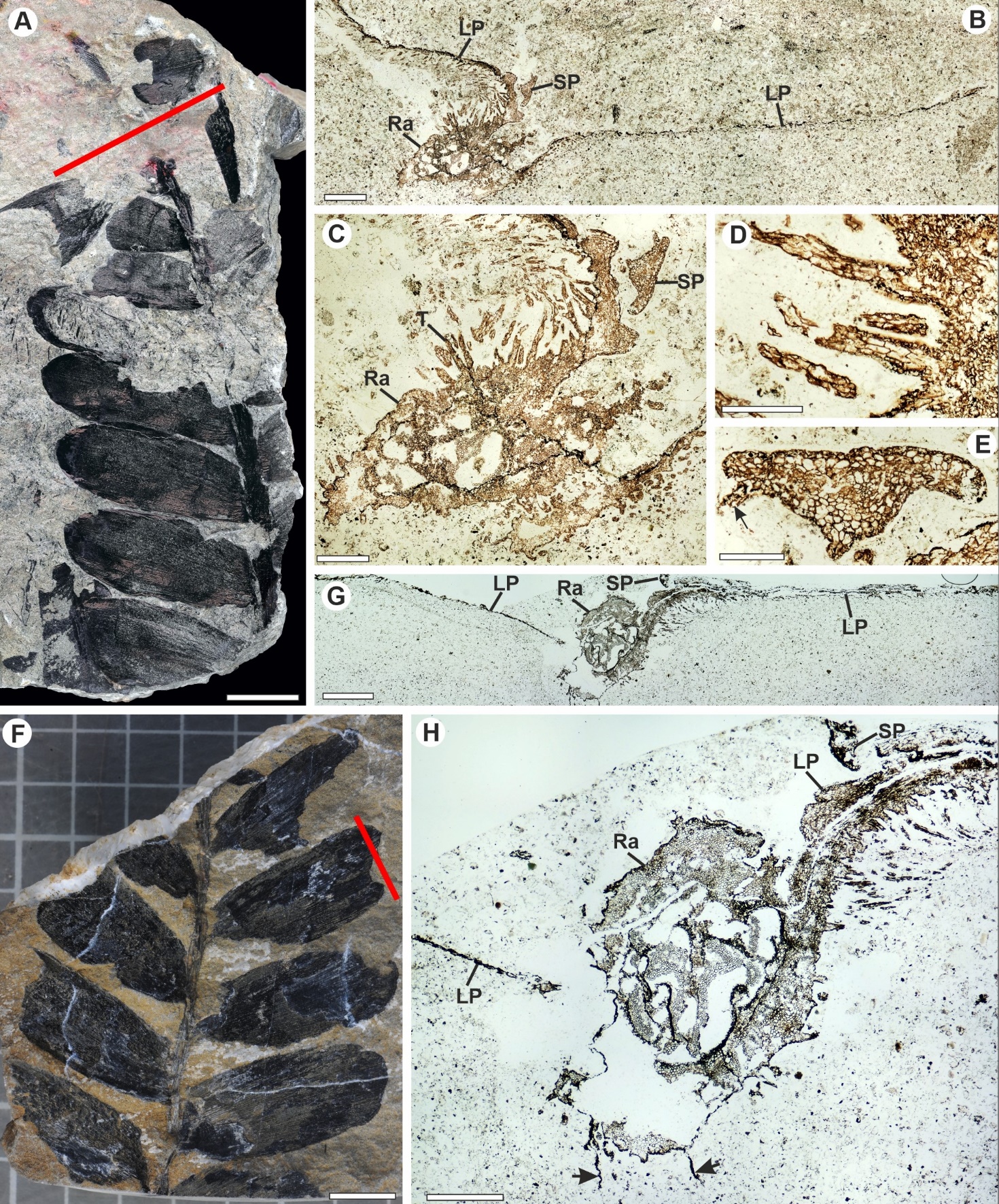


**Fig. S1.** *Paratingia fuyuanensis* sp. nov.

A–E. Specimen YNUPB11008. A. Hand specimen. Red line indicates the position of cross-section through the leaf. Scale: 2 cm. B. CS through leaf apex in Fig. S1A. Slide: YH0882. Scale: 1 mm. C. Enlargement of a part of Fig. S1B showing rachis and a small pinnule. Slide: YH0882. Scale: 0.5 mm. D. Longitudinal section through trichomes. Slide: YH0882. Scale: 200 μm. E. Small pinnule in CS with a trichome on its outer-facing side (arrow). Slide: YH0882. Scale: 200 μm. F–H. Specimen 72014. F. Hand specimen with red line indicating position of CS through a large pinnule. Scale: 1 cm. G. CS through leaf. Slide: WP2-0812. Scale: 2 mm. H. Enlargement of a part of Fig. S1G showing rachis and a partially preserved small pinnule. Arrows indicate trichomes on abaxial rachis surface. Scale: 1 mm. Abbreviations: CS–cross-section; LP–large pinnule; LS–longitudinal section; Ra–rachis; SP–small pinnule; T–trichome.

three parts: a lower or abaxial transversely or vertically elongated main part, an upper or adaxial narrow neck-like part, and the two outcurved (excurved) lateral margins. The distance between the ends of the two lateral margins represents the largest width of the inverted Ω-shaped xylem strand. In YNUPB11009, the main part of the inverted Ω-shaped xylem strand is usually more or less transversely elongated, whereas in specimens 72014 and YNUPB11008 it is mostly vertically elongated. The xylem strand of the main and neck-like parts is 3–5 tracheids thick and much thicker at the lateral margins. At the center of the lateral margins, there are one to two parenchymatous regions, which consist of protoxylem tracheids and parenchyma cells (Fig. S3B). The other parts of the xylem strand are all metaxylem tracheids, which are polygonal and isodiametric with the diameters mostly 25–35 μm (Fig. S3B). In longitudinal section, the area consisting of parenchyma cells and very small tracheids at the outcurved lateral margins of the xylem strand comprises longitudinally elongate parenchyma cells and tracheids with helical thickenings (Fig. 1G; Fig. S2E). Tracheids of the metaxylem possess scalariform thickenings (Fig. 1G).

The sclerenchymatous sheath surrounding the inverted Ω-shaped strand varies greatly in thickness from 2–3 to 8–9 cells thick and is commonly discontinuous due to secretory canals or diverging pinnule traces (Fig. S2A; Fig. S3B). Sclerenchyma cells possess thick walls and are isodiametric in cross-section and elongate in longitudinal section (Fig. S2E; Fig. S3B). Where preserved, fundamental tissue consists of parenchyma cells mixed with sclerenchyma cells. In longitudinal section, these parenchyma cells are elongated and possess minute pits on the walls.

Pinnule traces divergence and origination of small pinnules

Pinnule traces originate from the lateral margin of the large inverted Ω-shaped xylem strand and diverge successively from the upper (adaxial) side and the tip of the lateral margins. The trace

**
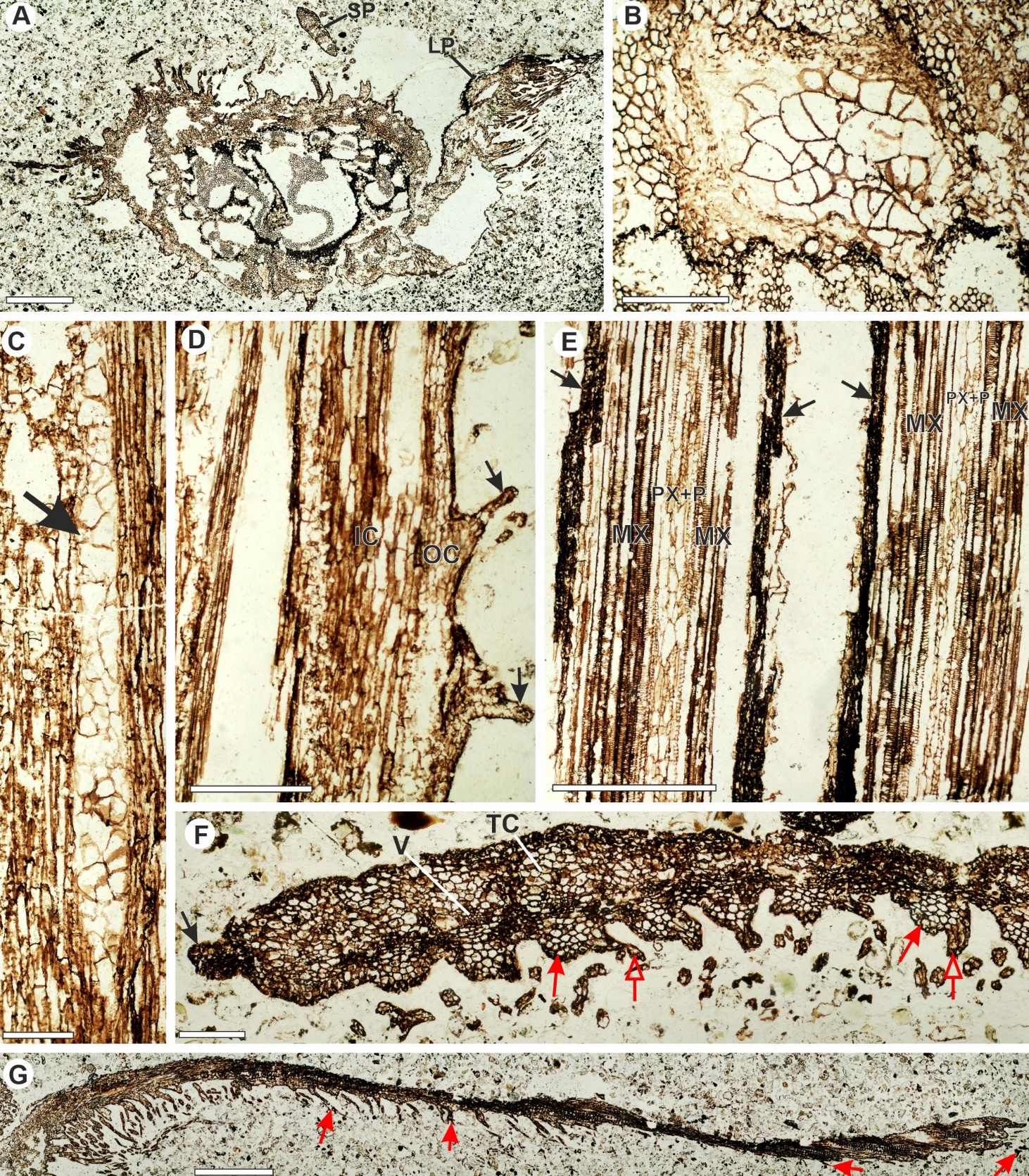
**

**Fig. S2.** *Paratingia fuyuanensis* sp. nov. Type specimen YNUPB11009.

A–B. CS of the rachis. A. Planoconvex rachis. Slide: YH0838. Scale: 1 mm. B. A large secretory canal filled with large parenchyma cells in the cortex. Slide: YH0879. Scale: 200 μm. C–E. LS of the rachis. C. A secretory canal filled with large parenchyma cells (arrow) in the cortex. Slide: YH0848. Scale: 200 μm. D. Trichomes (arrows) on the surface of the rachis and two-parted cortex. Slide: YH0851. Scale: 200 μm. E. Sclerenchymatous sheath (arrows) encircling the two lateral margins of the inverted Ω-shaped xylem strand, noting the area consisting of parenchyma and protoxylem. Slide: YH0851. Scale: 0.5 mm. F. Part of a large pinnule in CS, noting trichomes (red hollow arrows) on the abaxial ribs (red arrows). Black arrow indicates the base of a tooth at the lateral margin of the large pinnule. Slide: YH0825. Scale: 200 μm. G. A large pinnule in LS, noting abaxial trichomes (arrows) throughout. Slide: YH0855. Scale: 1 mm. Abbreviations: IC–inner cortex; MX–metaxylem; OC–outer cortex; P–parenchyma; PX–protoxylem; R–rib; TC–transfusion cells; V–vein.

diverges earlier from the upper (adaxial) side of the lateral margin (named trace A) and is closely followed by the trace diverging from the tip of the lateral margin (named trace B) (Fig. S4A–C). Traces A and B each have a small parenchymatous central area, with trace A usually arc-shaped or, in some cases, elliptical, whereas trace B is usually elliptical. After departing, trace A moves towards the adaxial side of the rachis, whereas trace B moves toward the abaxial side. Trace A divides several times rapidly to form 5–6 smaller arc-shaped traces (red arrows in Fig. S4D–I). However, trace B remains undivided for some distance before it divides to form 3–4 smaller arc-shaped traces (green arrows in Fig. S4G–I). The 8–10 smaller arc-shaped traces formed by trace A and B are located at the lateral side of the large inverted Ω-shaped xylem strand and arranged in an oblique straight line or in a curve paralleling the lateral sides of the rachis. Of these traces, the uppermost two or three, which are formed by trace A and are close to the adaxial side of the rachis (black arrow in Fig. S4I), enter the base of small pinnules, whereas the rest (ca. 6–8 in number) enter the base of large pinnules (Fig. S4I).

Small pinnules originate from a narrow and oblique split appearing at one flank of the upper (adaxial) surface of the rachis (black hollow arrows in Fig. S4B, F). Acropetally, the split moves toward the other flank and becomes gradually wider (purple arrows in Fig. S4C–E, G–H) and finally a protuberance rises (PB in Fig. S4F, I). This protuberance is the decurrent part of a small pinnule base. It is conical and leans toward the middle of the upper side of the rachis (PB in Fig. S4A, F, I; Fig. S6A). It has two sides of which one is further away from the middle of the upper side of the rachis and is termed the outer-facing side (OS); the other is close to the middle of the upper side of the rachis and is termed the inner-facing side (IS). The outer-facing side is the extension of the upper surface of the rachis and its surface is also rough and possesses trichomes (Fig. S6A). However, the inner-facing side is rather smooth and lacks trichomes. There are two veins in the protuberance. When the protuberance departs from the rachis it becomes the base of the small pinnule.


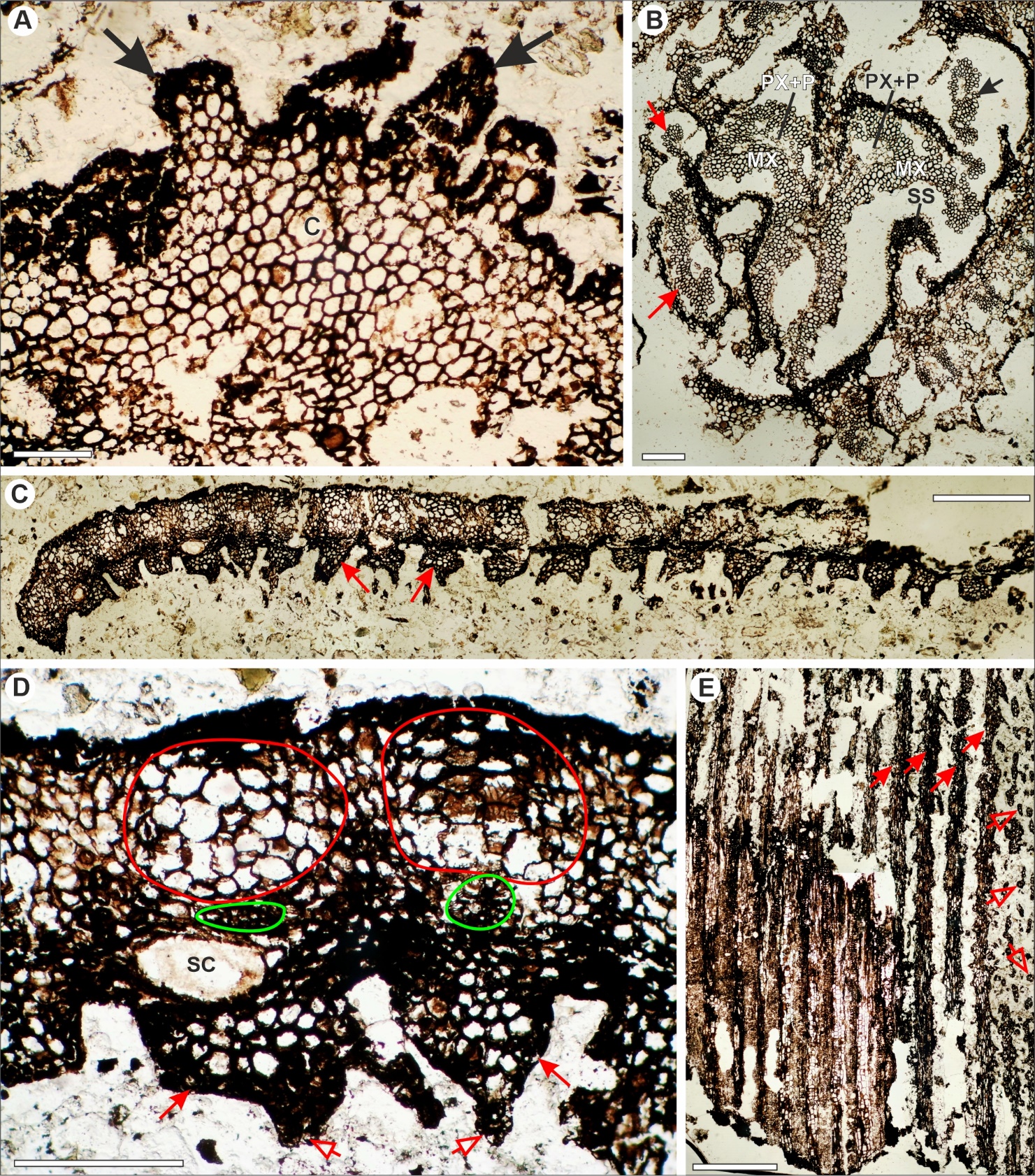


**Fig. S3.** *Paratingia fuyuanensis* sp. nov. Specimen 72014.

A, B. CS of the rachis. A. Trichomes (arrows) and cortex. Slide: WP2-0810. Scale: 100 μm. B. Inverted Ω-shaped xylem strand and pinnule traces (arrows). Slide: WP2-0811. Scale: 200 μm. C–E. Large pinnule. C. CS showing ribs (arrows) on the abaxial side. Slide: WP2-0829. Scale: 0.5 mm. D. CS showing transfusion tissue (red circles), very small veins (green circles), ribs (red arrows) and trichomes (red hollow arrows). Slide: WP2-0829. Scale: 200 μm. E. Paradermal section showing ribs (red arrows) and trichomes (red hollow arrows). Slide: WP2-0817. Scale: 1 mm. Abbreviations: C–cortex; SC–secretory cavity; SS–sclerenchymatous sheath.

**
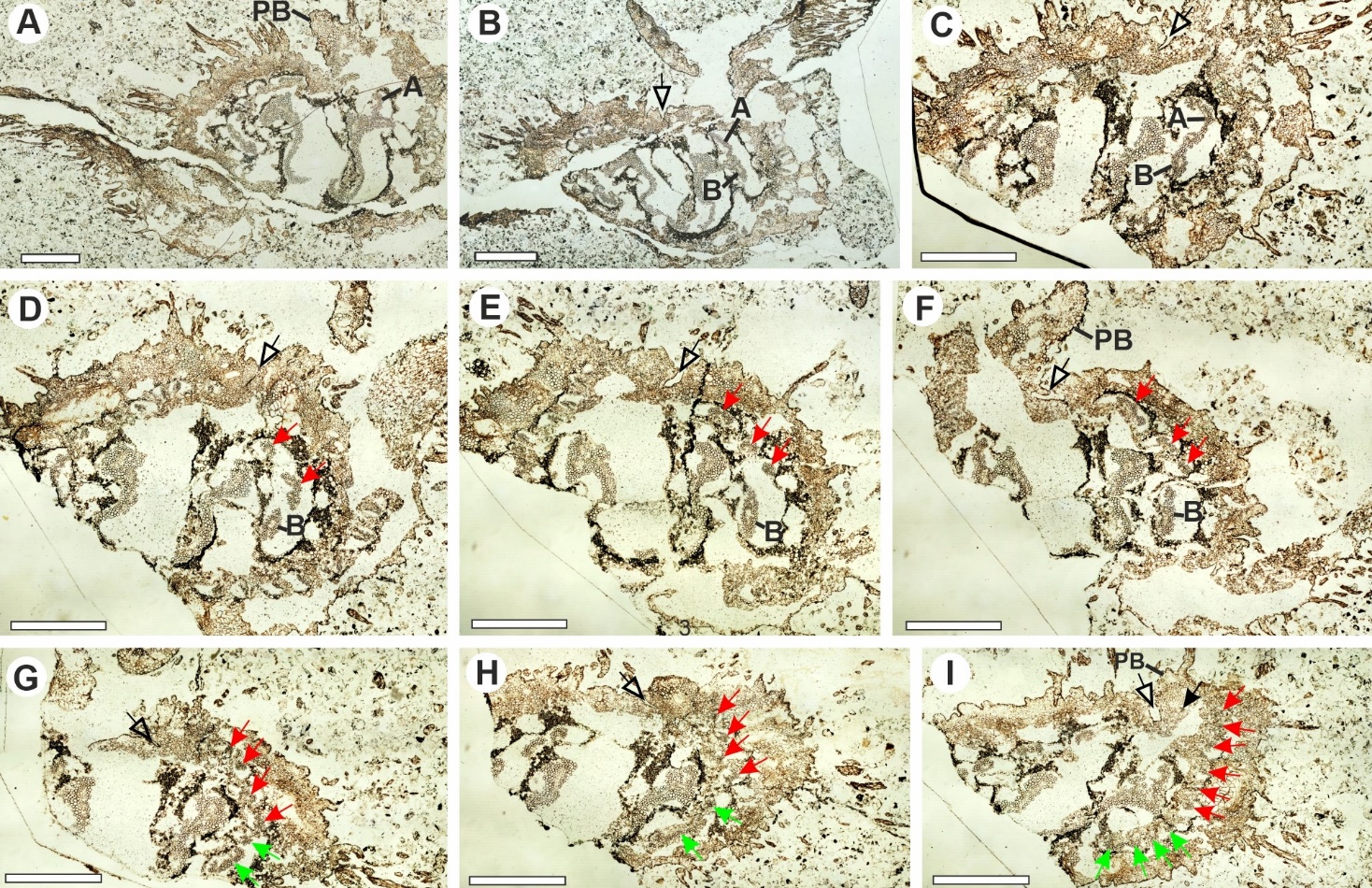
**

**Fig. S4.** *Paratingia fuyuanensis* sp. nov. Type specimen YNUPB11009.

Successive cross-sections of the rachis showing divergence of pinnule traces and the origination of a small pinnule. Black arrow in Fig. S4I shows a trace in the pinnule base. Full explanation is presented in the text. Slides: A–YH0888, B–YH0887, C–YH0855, D–YH0860, E–YH0862, F–YH0869, G–YH0872, H–YH0875, I–YH0881. Abbreviation: PB–pinnule base. Scales: 1 mm. A–Trace A, Red arrows–smaller arc-shaped of trace A, Green arrows–smaller arc-shaped of trace B.

Small pinnules

Successive cross-sections through the leaf in YNUPB11009 reveal that small pinnules are arranged in two rows and attached alternately on each flank of the upper (adaxial) rachis surface (Fig. S5). Based on changes in the shape and size of the small pinnule in successive cross-sections (Fig. S5; Fig. S6), it is deduced that small pinnules are probably elongated elliptical with maximum widths of 1.45–1.95 mm in the middle part, and are up to 10 mm long. Small pinnules are located directly above the rachis and extend acropetally in a parallel direction with the rachis. Surfaces of small pinnules are oblique or vertical to the upper surface of the rachis. Small pinnules taper in thickness apically and are 700–850 μm thick at the base (Fig. S6B), 300–470 μm in the middle (Fig. S6C) and 170–220 μm apically (Fig. S6E). The outer-facing side of the basal and middle parts of small pinnules are ribbed to different extents and possesses trichomes (Fig. 1I; Fig. S6B, C), whereas the inner-facing side is rather smooth and lacks ribs and trichomes. There are two or three veins in the basal part of small pinnules. Apically, small pinnules become gradually wider and possess more veins. Some veins are located at the margin of the small pinnule, which means that, in the small pinnules of this species, venation is radiate. In the middle part of different small pinnules there are three, four or up to six veins. From the middle part to the apex, small pinnules gradually become narrower and contains fewer veins. The apex may furcate into two or three short segments (lobes) and each segment contains a vein (Fig. 1K; Fig. S5I, O; Fig. S6F). Sporadic teeth extend from the lateral margins of the middle and upper parts of small pinnules. They are very short with lengths typically less than 0.3–0.4 mm. In cross-section, teeth are nearly round and thin with the diameters less than the pinnule thickness. They consist of small and thick-walled cells and lack veins (Fig. S5D, G, M; Fig. S6D, G).

The epidermis of small pinnules is uniseriate and cells vary in sizes and shapes in cross-section. Most epidermal cells are sub-circular though some are rectangular. Veins consist of a primary xylem strand containing several or more than 10 small tracheids with diameters of 10–15 μm. The protoxylem is located at the center of the primary xylem strand (Fig. 1I, J; Fig. S6G, H). Phloem is difficult to recognize. Veins connect the epidermis of the inner- and outer-facing sides of the small pinnules by transfusion tissue and thick-walled parenchymatous tissue respectively. Transfusion tissue at the basic part of small pinnules is 2–3 cells-wide (Fig. S6B), but apically it increases in width and semi-encircles veins at the pinnule apex (Fig. S6D, E). Transfusion cells are polygonal and isodiametric with diameters of 25–40 μm in cross-section and possess reticulate wall pitting (Fig. 1I, J). Thick-walled parenchymatous tissue comprises up to 8–10 layers of cells, which are slightly smaller than transfusion cells and forms significant ribs on the outer-facing side at the basic part of small pinnules (Fig. 1I, J; Fig. S6B, C). However, apically, thick-walled parenchymatous tissue decreases in the thickness, only 3–4 layers of cells thick, and does not form significant ribs (Fig. S6D, E). Mesophyll is differentiated into parenchymatous and sclerenchymatous tissues, which connect the outer-facing side and the inner-facing side of small pinnules respectively. Parenchymatous mesophyll cells are sub-circular with diameters of 25–45 μm or vertically elongated


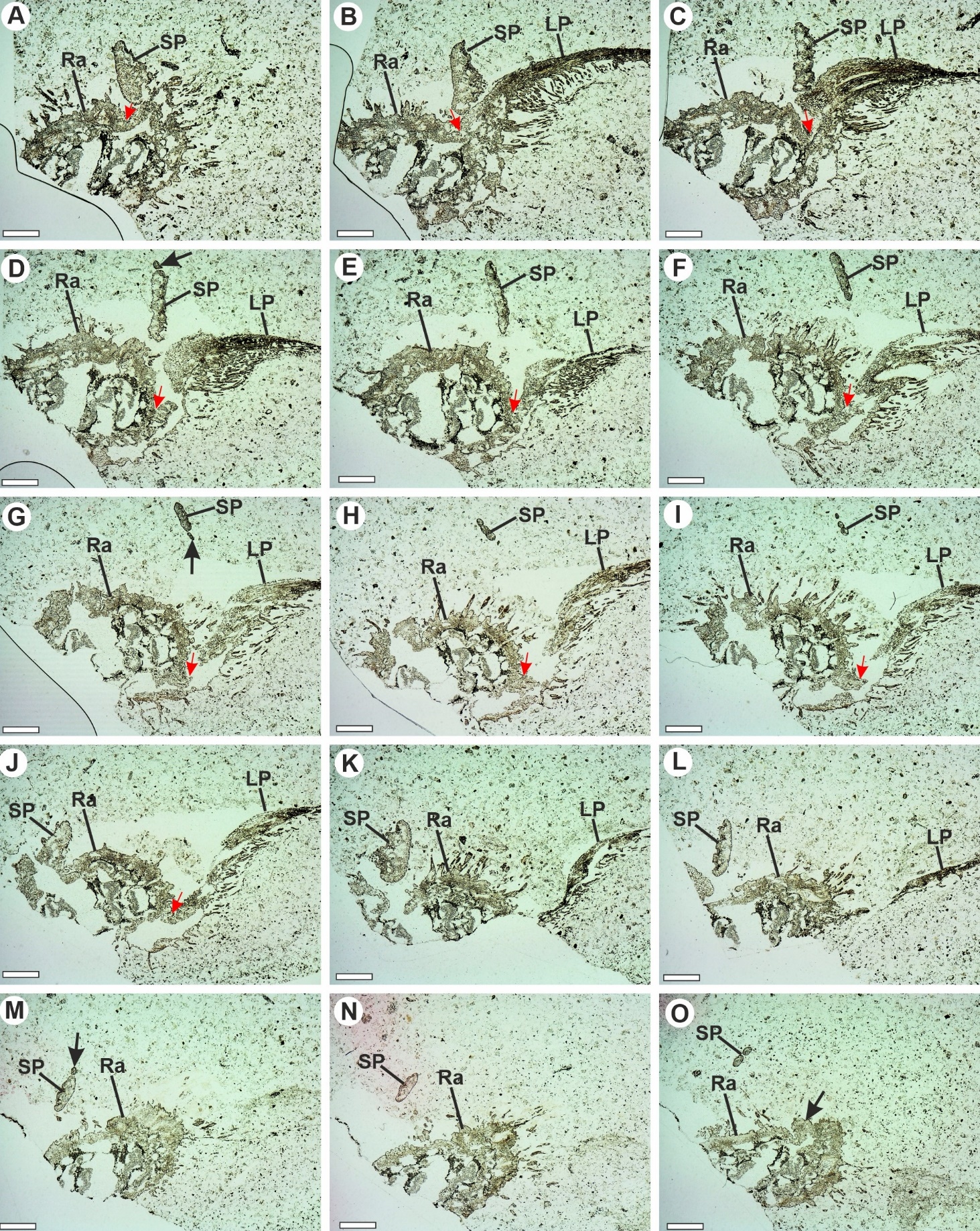


**Fig. S5.** *Paratingia fuyuanensis* sp. nov. Type specimen YNUPB11009.

Acropetal successive cross-sections of the leaf showing two small pinnules attaching alternately on each flank of the upper surface of the rachis and extend in a parallel direction with the rachis. Note that the apices of these two small pinnules furcate into two (Fig. S5O) or three (Fig. S5I) short segments. Black arrows indicate teeth (Fig. S5D, G, M) or pinnule base (Fig. S5O). Red arrows indicate the attachment point of large pinnules to the rachis. Slides: A–YH0854, B–YH0855, C–YH0858, D–YH0860, E–YH0861, F–YH0863, G–YH0865, H–YH0866, I–YH0867, J–YH0869, K–YH0870, L–YH0873, M–YH0875, N–YH0877, O–YH0879. Scales: 1 mm.


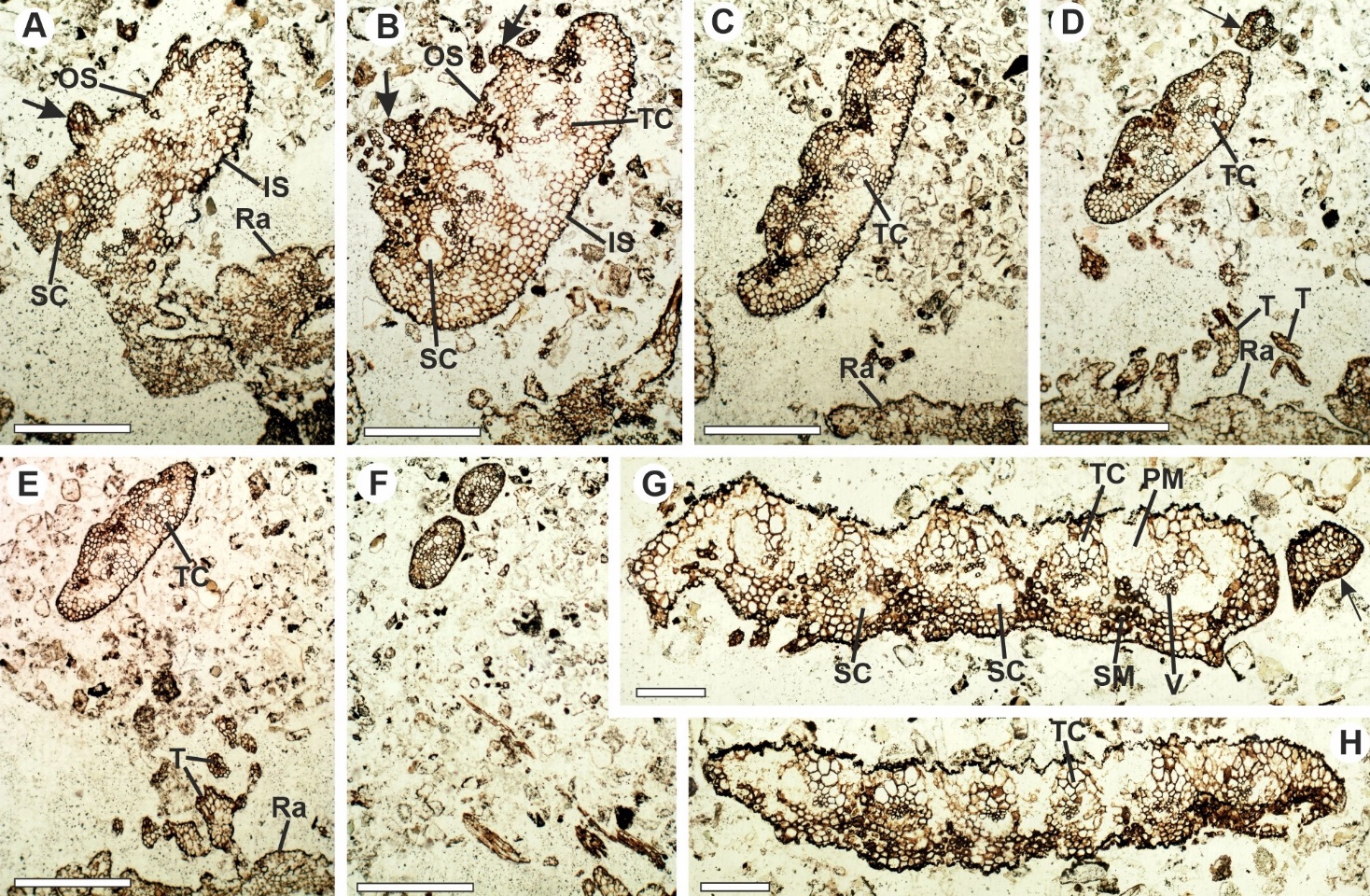


**Fig. S6.** *Paratingia fuyuanensis* sp. nov. Type specimen YNUPB11009.

A–F. Successive cross-sections of a small pinnule from base to apex. Arrows indicate trichomes (Fig. S6A, B) and a tooth (Fig. S6D). G–H. CS of two pinnules with five (Fig. S6G) and six (Fig. S6H) veins. Arrow in Fig. S6G indicates a tooth. Abbreviations: IS–inner-facing side; OS–outer-facing side; PM–parenchymatous mesophyll; SM–sclerenchymatous mesophyll. Slides: A–YH0869, B–YH0870, C–YH0873, D- YH0875, E–YH0877, F–YH0879, G–YH0860, H–YH0861. Scales: 0.5 mm (A–F); 200 μm (G–H).

with lengths up to 60 μm (Fig. 1I, J; Fig. S7G). In some places, parenchymatous mesophyll is poorly preserved or destroyed due to its very thin cell walls (Fig. S6G, H). Sclerenchymatous mesophyll cells are isodiametric, very small, with the diameters from 15–25 μm, and have very thick cell walls that are up to 7–8 μm thick. Secretory cavities can be seen in some cross-sections and are mostly located in thick-walled parenchymatous tissue. They are almost circular or elliptical in cross-section and elongate in longitudinal section of small pinnules and some of them contain secretory products (Fig. 1I, J, Fig. S6A–C, G; Fig. S7E).

Large pinnules

When examining successive acropetal cross-sections of the rachis, the attachment point of large pinnules to the rachis is firstly on the upper surface of the rachis by its lower margin (Fig. S5A, B; Fig. S7H). Then, the attaching point moves to the lateral side and at last to the lower surface of the rachis by the upper margin of large pinnules (Fig. S5C–J; Fig. S7I–K). In this arrangement, bases of large pinnules are attached to the lateral side of the rachis and inclined forward, but twist backwards to spread the blade in a horizontal plane. Anatomical features of large pinnules in cross-section are similar to those of the small pinnules. There are also minute teeth along the lateral sides of large pinnules (Fig. S2F). The upper (adaxial) surface of large pinnules is comparable to the inner-facing surface of small pinnules and is flat and lacks ribs, whereas the lower (abaxial) surface is comparable to the outer-facing surface of small pinnules and is strongly undulated because of well-developed ribs (Fig. 1H; Fig. S2F; Fig. S3C, D). The ribs bear are abundant multiseriate multicellular trichomes. In large pinnules of specimen 72014, in longitudinal and paradermal sections, cells of the transfusion tissue are rectangular or polygonal with well-developed reticulate pitting (Fig. S7B–F). Primary xylem tracheids are long and thin (Fig. S7F). Cells of thick-walled parenchymatous tissue are longitudinally elongate with tapered ends (Fig. S7A, F). Parenchymatous mesophyll differentiates into palisade and spongy tissues (Fig. 1H; Fig. S7G). In longitudinal and paradermal sections, sclerenchymatous mesophyll cells are isodiametric (Fig. 1H, L; Fig. S7G).

**
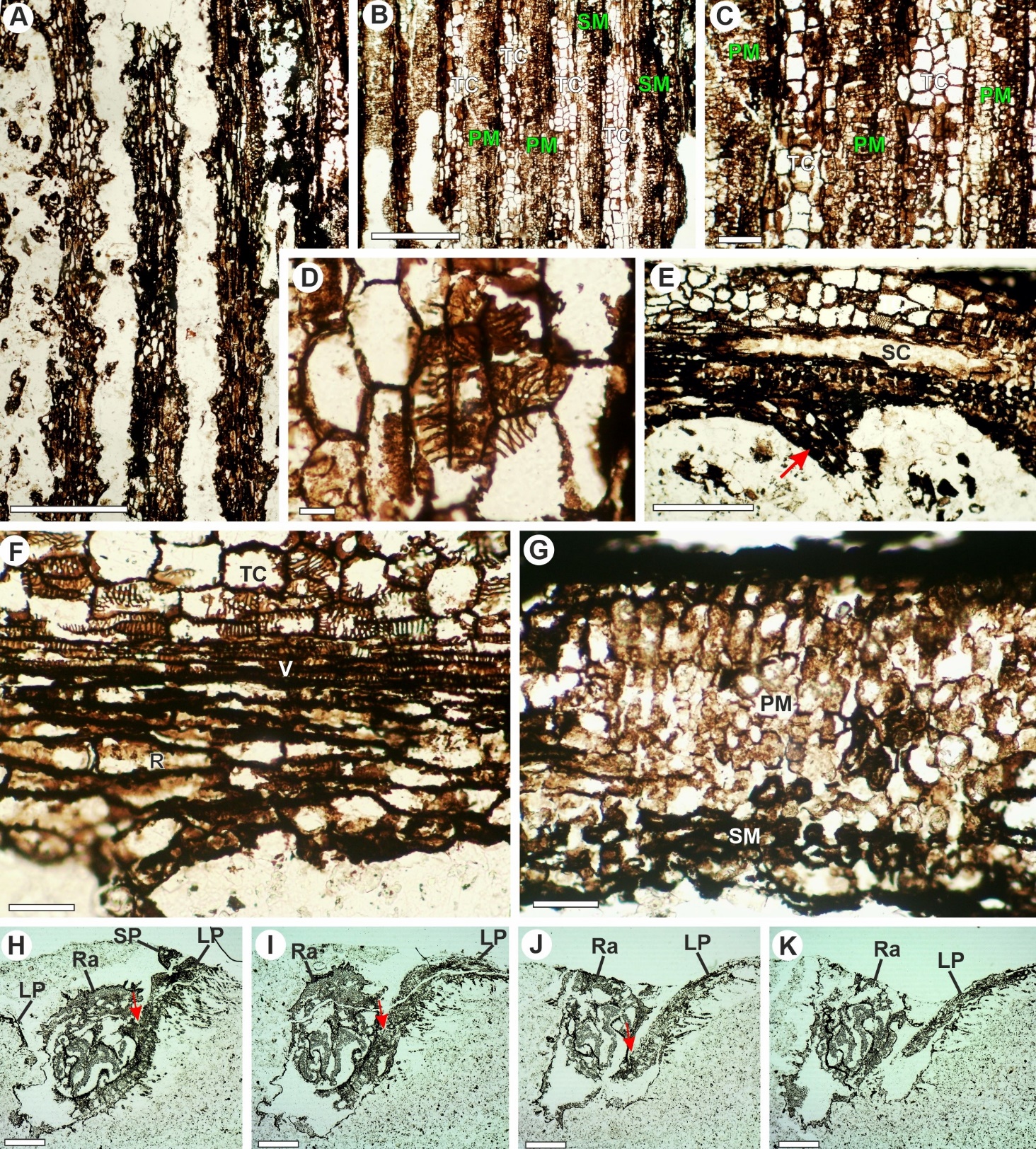
**

**Fig. S7.** *Paratingia fuyuanensis* sp. nov. Specimen 72014.

A–D. Paradermal sections of large pinnule. Slide: WP2-0817. A. Ribs on the abaxial side. Scale: 0.5 mm. B. Transfusion cells, parenchymatous mesophyll and sclerenchymatous mesophyll. Scale: 0.5 mm. C. Enlargement from Fig. S7B. Scale: 100 μm. D. Reticulate pitting on transfusion cell walls. Scale: 20 μm. E–G. Longitudinal sections of large pinnule. E. Secretory cavity and trichome (arrow). Slide: WP2-0822. Scale: 200 μm. F. Large transfusion cells with reticulate pitting, very narrow tracheids of the vein and elongate thick-walled cells of the rib. Slide: WP2-0821. Scale: 50 μm. G. Parenchymatous mesophyll (PM) and sclerenchymatous mesophyll (SM). Slide: WP2-0822. Scale: 50 μm. H–K. Successive cross-sections of the leaf showing attaching point (arrows) of large pinnules to the rachis moving from the upper surface (Fig. S7H) to the lateral side (Fig. S7K). Slides: WP2-0811, WP2-0810, WP2-0808, WP2-0807. Scales: 1 mm.

**Species** *Paratingia qingyunensis* Qin, He, et Wang sp. nov.

**Morphological and epidermal features**

This species is based on two specimens, YNUPB11007 and YNUPB11010. Specimen YNUPB11007 has morphological and anatomical preservation and is a mono-pinnate compound leaf with the preserved length of ca. 9 cm, whereas YNUPB11010 is anatomically preserved and its morphology is largely unknown.

In YNUPB11007 the rachis has a stable width of about 6 mm throughout the preserved length and represents the mid-part of the leaf lacking the base and apex. It has a row of large pinnules obliquely and alternatively attached to each lateral side of the rachis at angles of 70°–80° to the rachis (Fig. 3A). On the rock surface exposing the leaf, small pinnules are not observed because they are buried in the matrix. Large pinnules are elliptical and about 5 cm long and about 1.5 cm wide in the middle part. The length:width ratio of large pinnules is about 3.3:1. They taper to the apex and base and have a rather broad, semi-amplexicaul base and a sub-rounded or obtuse apex (Fig. 3A; Fig. 4L; Fig. S8A). Pinnule laminae do not spread out in one plane but form an angle with the bedding-plane, thus their lower margins are typically embedded in the matrix. Margins and apices of large pinnules are entire and lack teeth (Fig. 4L; Fig. S8A). Several veins extend from the pinnule base to the margins and apex, with ca. 30 veins per centimeter in the middle of large pinnules.

A small area preserving the epidermis was found from the abaxial surface near the base of a large pinnule of Specimen YNUPB11007 and was observed by stereoscopic microscopy and scanning electron microscopy (Fig. 3A–C; Fig. S8B). The epidermis lacks distinct costal and intercostal zones. Epidermal cells are rectangular or longitudinally polygonal, 25–75 μm long and 20–25 μm wide (Fig. 3C; Fig. S8B). Outer periclinal cell walls are more or less arching and filiform, so the surface of the epidermal cells looks coarse (Fig. 3C; Fig. S8B). Stomatal apparatuses are randomly distributed and do not form rows (Fig. 2B, C; Fig. 3A; Fig. 4C; Fig. S8B) but are almost equi-spaced. Each stomal apparatus consists of

**
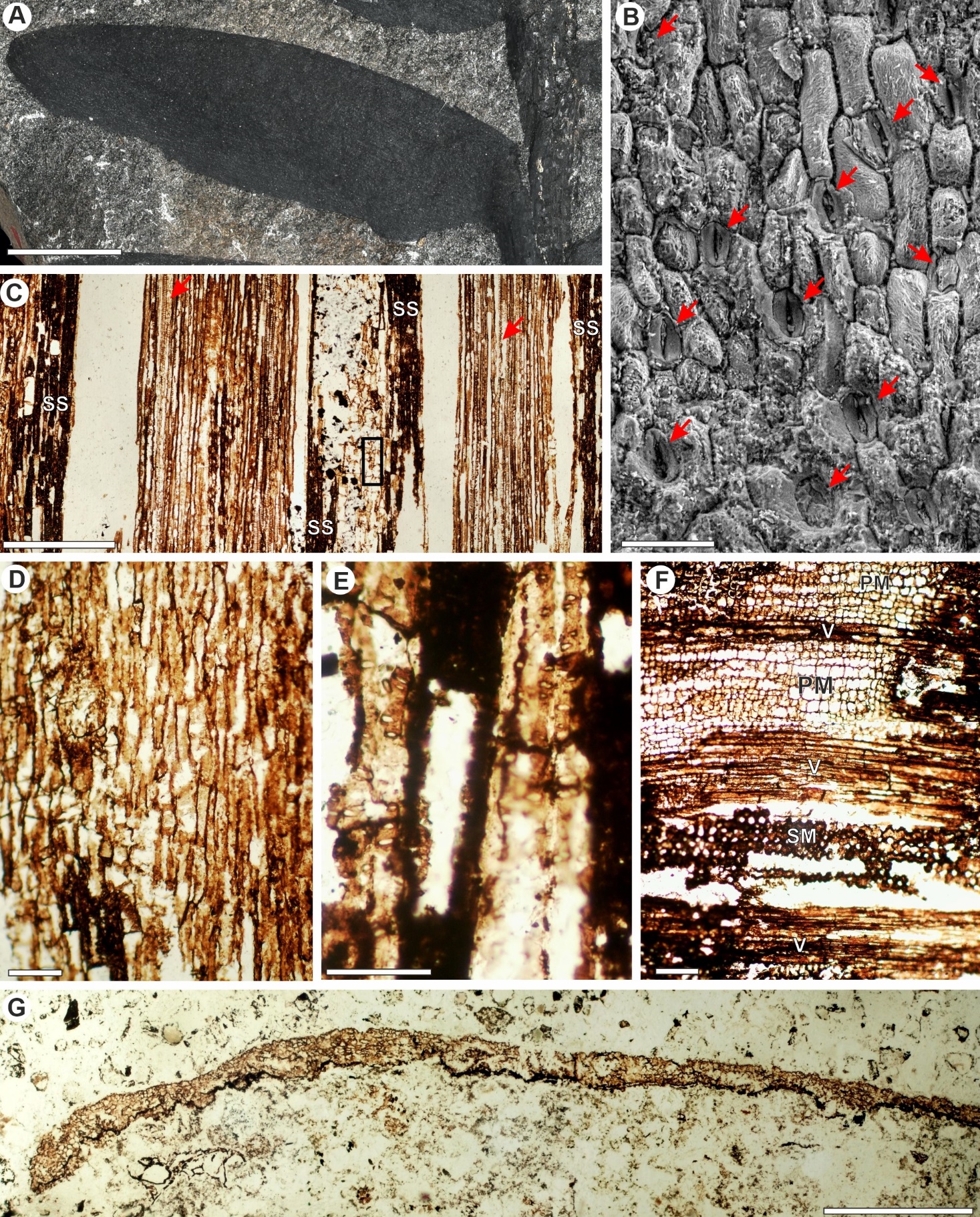
**

**Fig. S8.** *Paratingia qingyunensis* sp. nov. Type specimen YNUPB11007.

A. A large pinnule. Scale: 1 cm. B. SEM of the abaxial epidermis of large pinnule showing several stomatal apparatuses (arrows). Scale: 50 μm. C–E. Longitudinal section of the rachis. C. Lateral margins of the inverted Ω-shaped xylem strand, with arrows indicating protoxylem. Scale: 0.5 mm. D. Cells of the cortex. Scale: 100 μm. E. Enlargement of the area in black frame of Fig. S8C showing minute pits on cell walls of the fundament tissue. Scale: 50 μm. F. Paradermal section of the large pinnule. Scale: 100 μm. G. Cross-section of a large pinnule. Scale: 0.5 mm. Slides: C–YH0769, D–YH0768, F–YH0778, G–YH0779.

two slightly sunken guard cells and is oval in outline and ca. 28 μm long and ca. 17 μm wide. Guard cells are reniform, ca. 28 μm long and 6–8 μm wide, with a smooth outer surface. Stomatal apertures are narrowly fusiform with their long axes pointing in a uniform direction and parallel to the epidermal cells’ long axes (Fig. 3C; Fig. 6C; Fig. S8B).

YNUPB11010 is an anatomically preserved leaf and its morphological features are unknown as it was entirely encased in the matrix.

**Anatomical features**

Rachis

The cross-sectional shape of the rachis is planoconvex with its upper (adaxial) side roughly flat and the lower (abaxial) side convex. The rachis is ca. 5 mm wide and 2.7 mm high in both specimens. The surface of the rachis is slightly undulated but smooth, lacking hairs or trichomes (Fig. 3D, E, F; Fig. S9; Fig. S11B; Fig. S12B). The epidermis is unicellular and most cells are mostly slightly radially elongate or column-like, though in some places they are nearly isodiametric in cross-section with diameters of 10–18 μm. Epidermal cells possess thicker walls than those of cortical cells and their outer periclinal wall is extremely thickened and even papillary (Fig. 3H; Fig. S12C). The cortex is 7–10 cells (100–300 μm) thick in YNUPB11007 but can be more than 30 cells (up to 500 μm) thick in YNUPB11010. Cells of the cortex are parenchymatous and nearly isodiametric and polygonal in cross-section, with the diameters of 20–35 μm (Fig. 3G, H; Fig. S12C). In longitudinal section, cells of cortex are elongate or rectangular and 80 μm to more than 200 μm long (Fig. S8D). In the center of the rachis, a large vascular bundle is surrounded by poorly preserved fundamental tissue. The vascular bundle consists of a single long and continuous primary xylem strand without phloem preserved. In cross-section of the rachis, the xylem strand is an inverted Ω-shape with two outcurved (excurved) lateral margins (Fig. 3F; Fig. S12B). However, in some cross-sections of the rachis, the xylem strand is somewhat U-shaped (Fig. 3D; Fig. S9). The xylem strand is only 3–4 tracheids thick at its main part but increases in thickness toward the two outcurved (excurved) lateral margins where

**
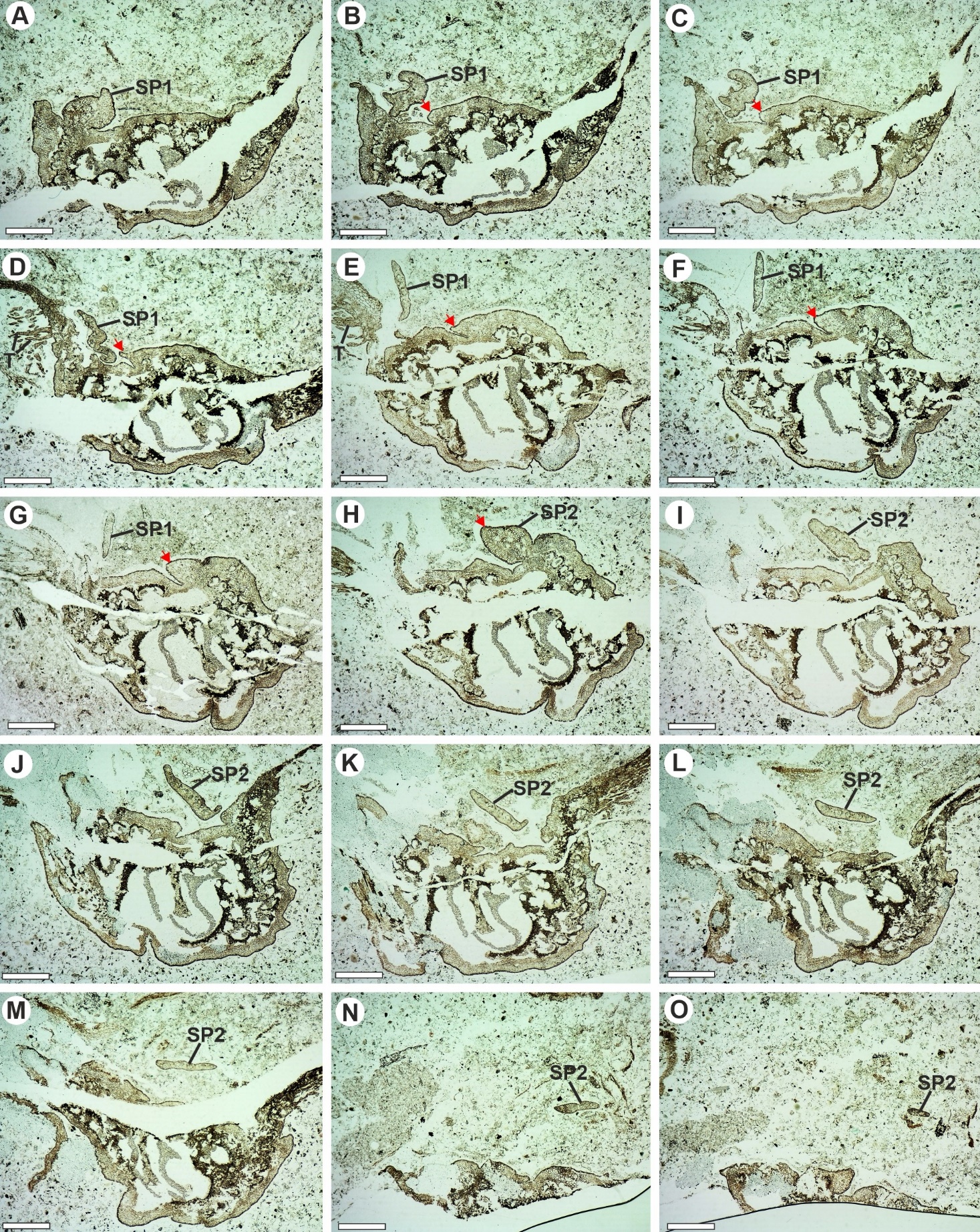
**

**Fig. S9.** *Paratingia qingyunensis* sp. nov. Type specimen YNUPB11007.

Successive cross-sections through the leaf showing two small pinnules (SP1 and SP2) attached alternately on each flank of the upper surface of the rachis and extending acropetally and parallel with the rachis. Red arrows indicate the origination of small pinnules. Slides: A–HY0781, B–HY0784, C–HY0785, D–HY0786, E–HY0788, F–HY0789, G–HY0789’, H–HY0790, I–HY0792, J–HY0796, K–HY0798, L–HY0800, M–HY0801, N–HY0803, O–HY0805. Scales: 1 mm.


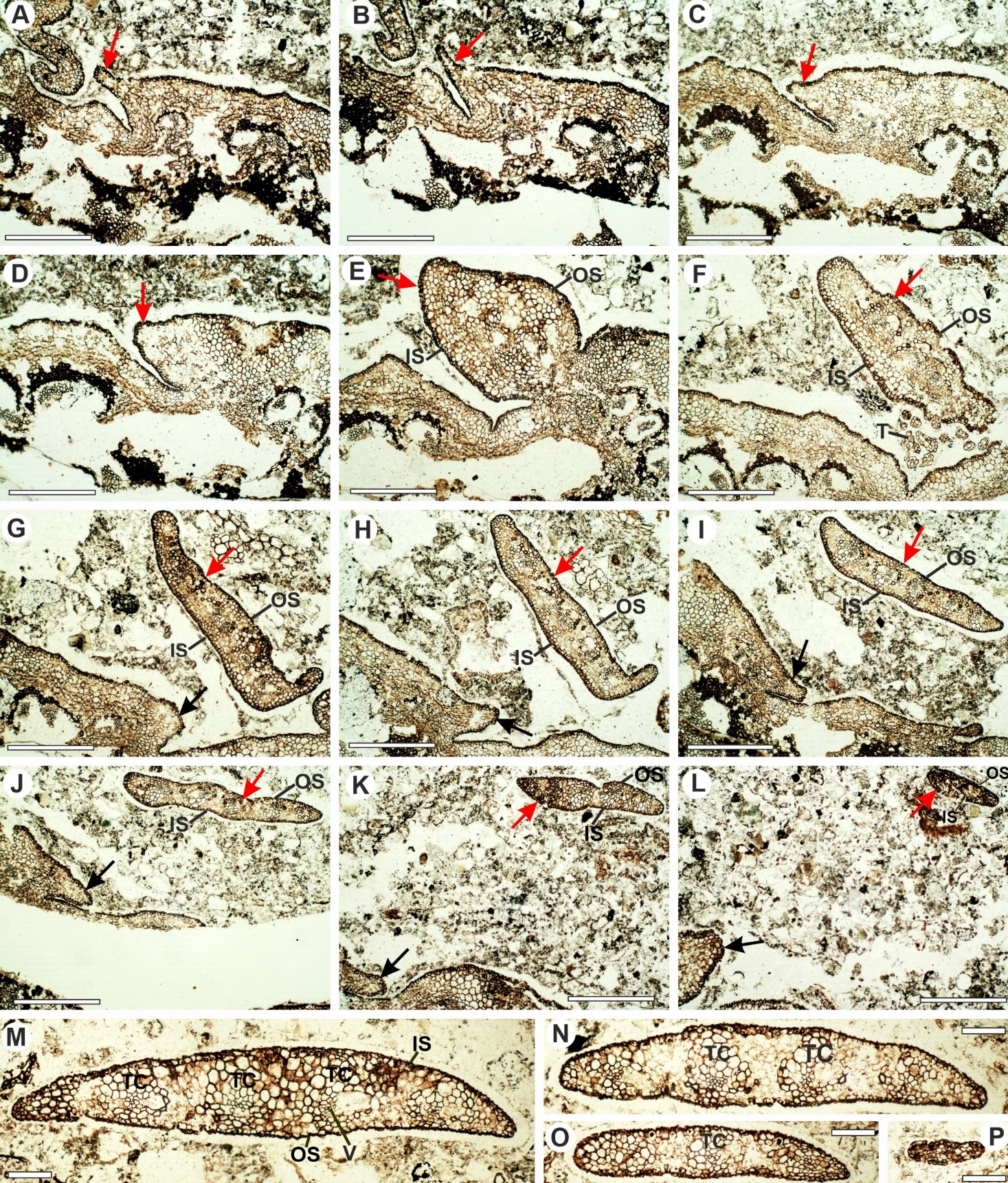


**Fig. S10.** *Paratingia qingyunensis* sp. nov. Specimen YNUPB11007.

A–L. Acropetal successive cross-sections through the rachis showing development of a small pinnule. A–E. A protuberance (red arrows) is forming on the adaxial side of the rachis. F. The protuberance is departing the rachis and becomes the base of a small pinnule. F–L. Acropetal successive cross-sections of the small pinnule (red arrows) from its base (Fig. S10F) to apex (Fig. S10L). Note that the apex is entire. From Fig. S10G to Fig. S10L another protuberance (black arrows) is forming which will become another small pinnule. M–P. Acropetal successive cross-sections of another small pinnule from the mid region (Fig. S10M) to apex (Fig. S10P). Slides: A–HY0786, B–HY0787, C–HY0788, D–HY0789, E–HY0790, F–HY0792, G–HY0796, H–HY0797, I–HY0800), J–HY0801, K–HY0803, L–HY0805, M–HY0815, N–HY0815, O–HY0816, P–HY0816. Slides: 0.5 mm (A–L), 100 μm (M–P).

it is up to 10 tracheids thick (Fig. 3I; Fig. S12B). The xylem strand is surrounded by a sclerenchymatous sheath up to 6–7 cells thick and comprising thick-walled cells. Secretory cavities are distributed along the sheath. At the outcurved lateral margins of the xylem strand there are one or two areas consisting of parenchyma cells and very small tracheids, which represent protoxylem (Fig. 3I). The other parts of the xylem strand comprise polygonal and isodiametric metaxylem tracheids with diameters of 25–35 μm. In longitudinal section through the lateral margins of xylem strand, the areas consisting of parenchyma cells and protoxylem at the outcurved lateral margins of the xylem strand are more easily recognized as they comprise longitudinally elongate parenchyma cells and tracheids with helical thickenings (Fig. 3J). Tracheids of the metaxylem possess scalariform thickenings (Fig. 3J). Where preserved, fundamental tissue around the vascular bundle consists of parenchyma cells mixed with sclerenchyma cells. In longitudinal section, these parenchyma cells are elongated and possess minute pits on cell walls (Fig. S8E).

The origin and development of the pinnule traces of this species is the same as that of *Paratingia* *fuyuanensis* sp. nov. based on the successive cross-section of rachis of specimen YNUPB11007; this information is not repeated here.

Small pinnules

Successive cross-sections of the rachis of specimen YNUPB11007 were made to reveal the arrangement of small pinnules on the rachis and the changes of anatomical features of small pinnules from their base to the apex. This series of cross-sections contain four small pinnules and they are arranged in two rows and attached alternatively on each flank of the upper (adaxial) rachis surface (Fig. S9). Small pinnules also originate from a protuberance on the upper (adaxial) surface of the rachis in the same way as in specimen YNUPB11009 of *Paratingia fuyuanensis* sp. nov. (Fig. S10A–E). They extend forward along the rachis or at a very small angle to the rachis. Their surface is oblique to (Fig. S9B–G) or nearly parallel to (Fig. S9I–O) the upper (adaxial) side of the rachis. Small pinnules are ca. 6–7 mm long in specimen YNUPB11007.

**
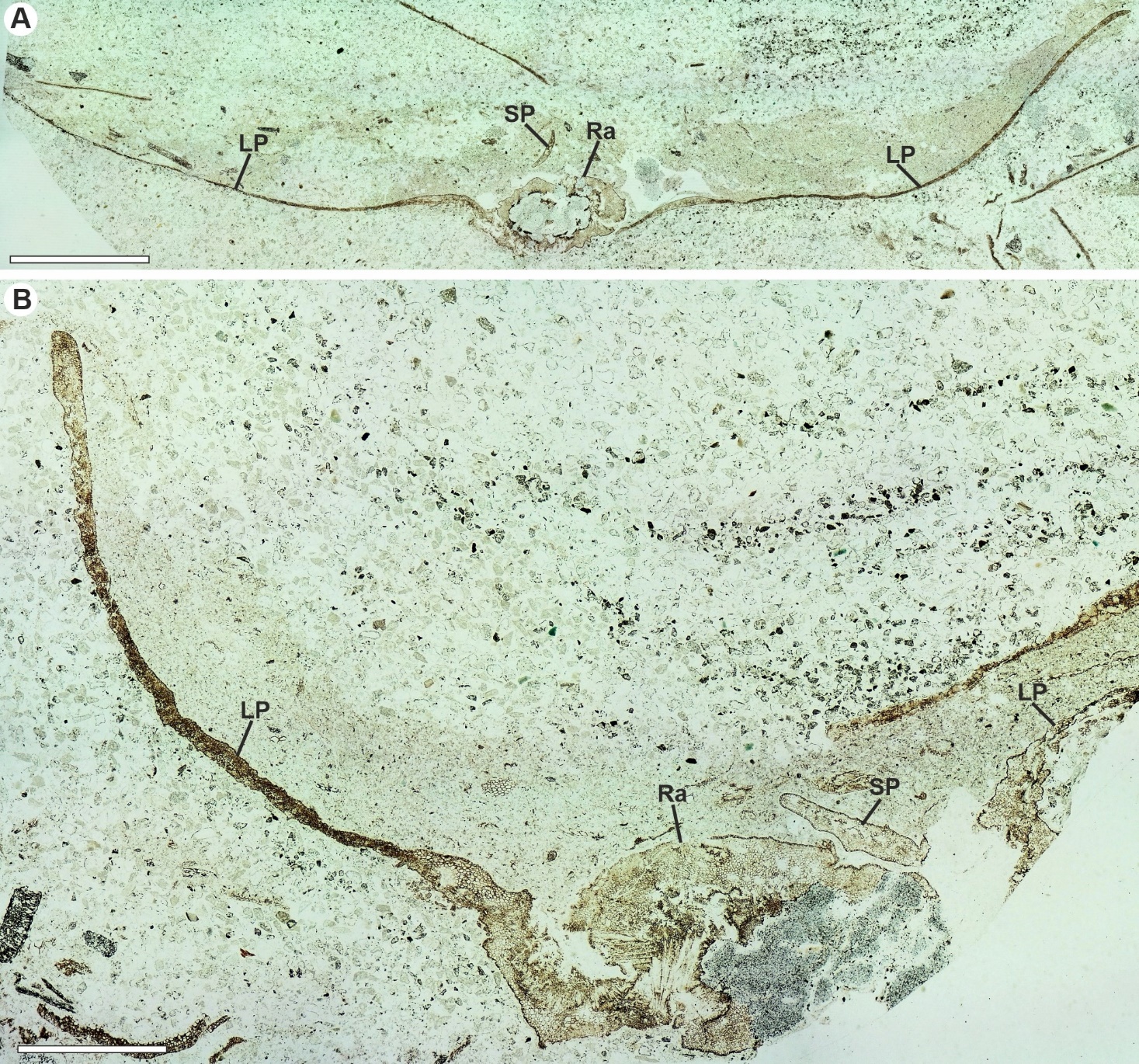
**

**Fig. S11.** *Paratingia qingyunensis* sp. nov. Specimen YNUPB11010.

A, B. Two cross-sections of the leaf. Slides: A–HY0886, B–HY0885. Scales: 5 mm (A), 2 mm (B).

**
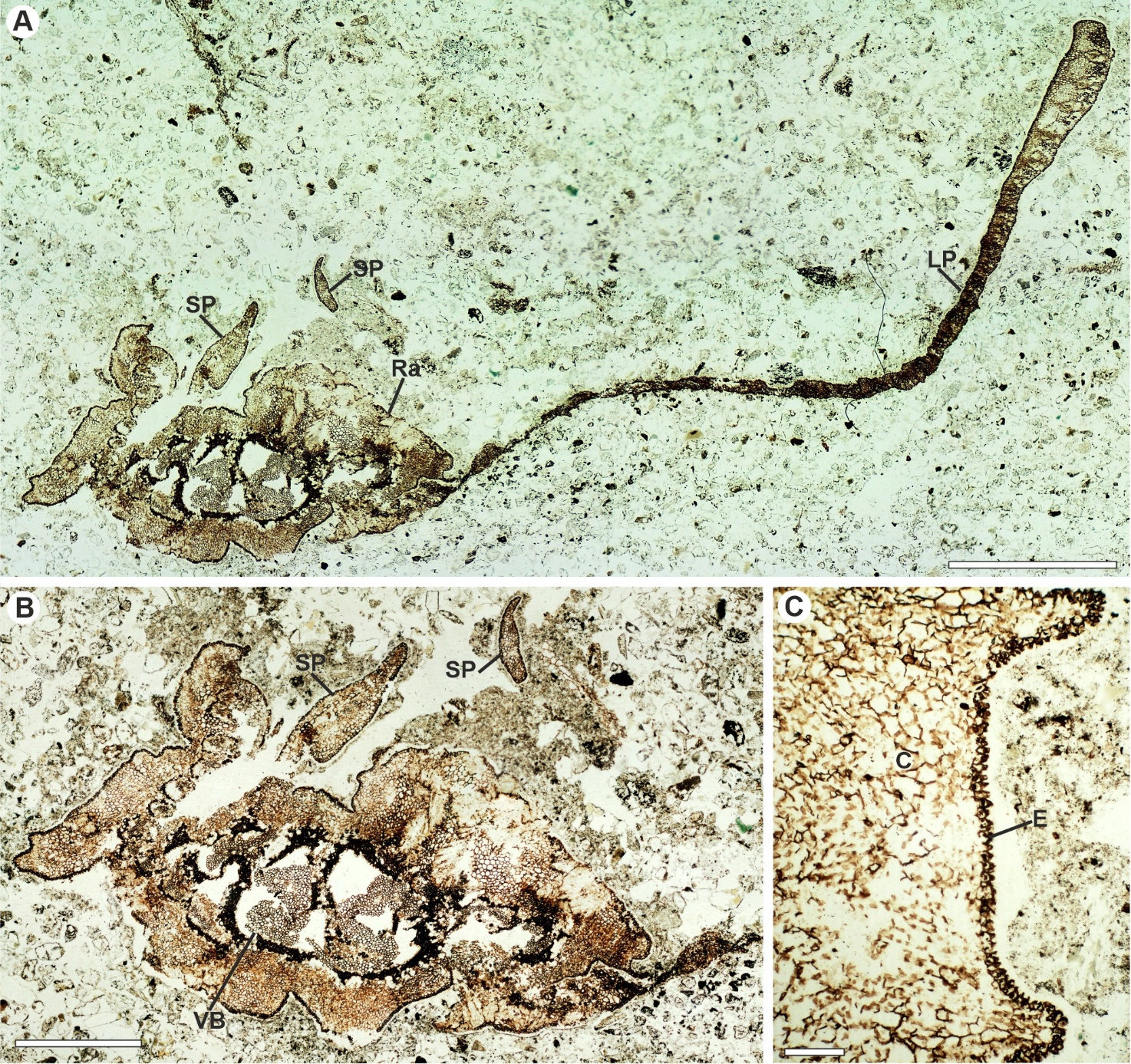
**

**Fig. S12.** *Paratingia qingyunensis* sp. nov. Specimen YNUPB11010.

A. Another cross-section of the leaf. Slide: HY0884. Scale: 2 mm. B. Enlargement of a part of Fig. S12A, showing the rachis and two small pinnules. Scale: 1 mm. C. Part of the rachis in cross-section, showing cortex (C) and epidermis (E). Slide: HY0886. Scale: 100 μm.

The length of small pinnules of specimen YNUPB11010 is unknown due to the lack of successive cross-sections of the leaf. The basal part of small pinnules of the specimen YNUPB11007 is usually folded in varying degrees, which makes the cross-sectional shape of small pinnules undulated (Fig. S9A–D), but the middle and upper parts are usually flat. The surface of the pinnule is usually smooth and lacks ribs and trichomes, except for the basal part, which commonly bears

**
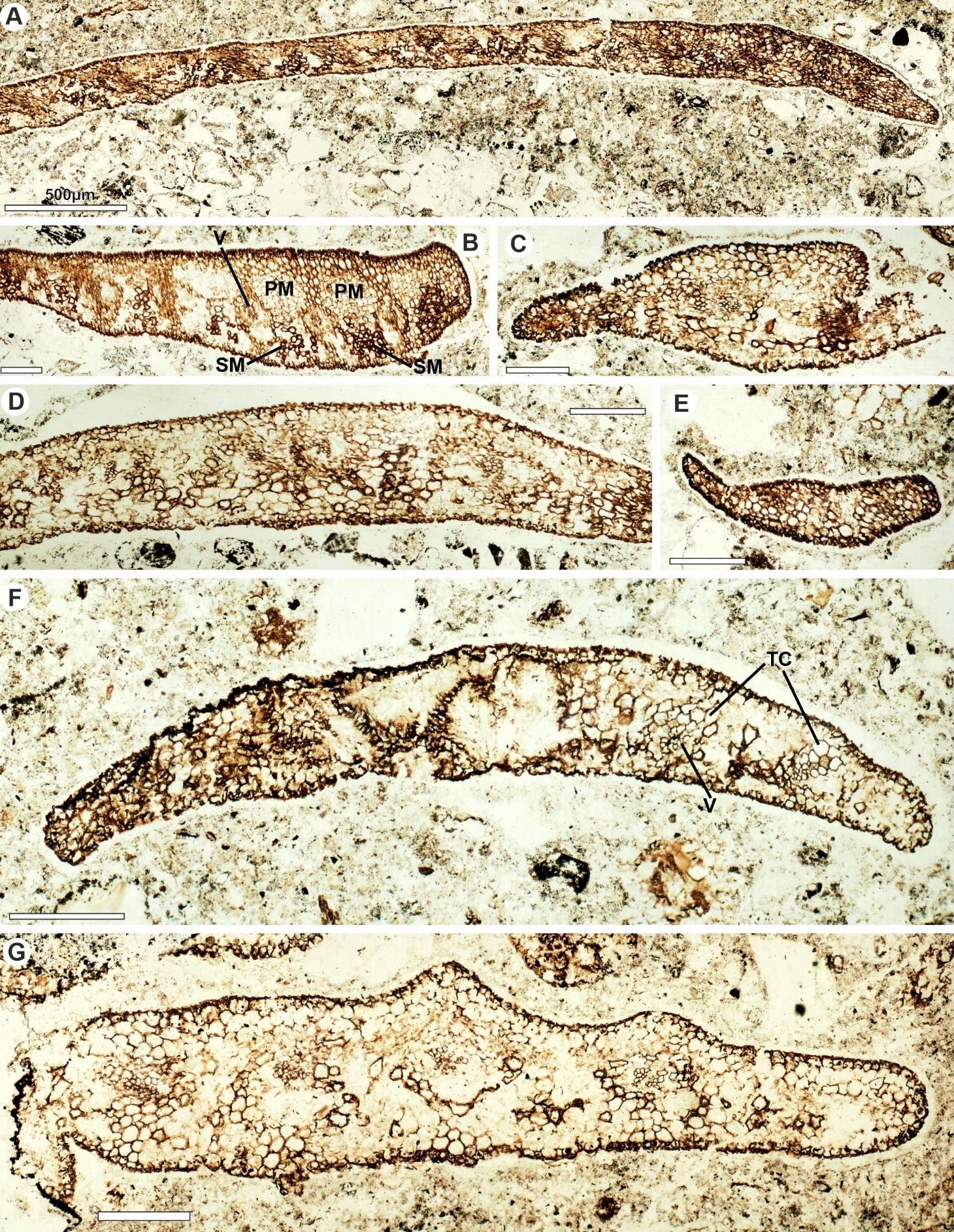
**

**Fig. S13.** *Paratingia qingyunensis* sp. nov. Specimen YNUPB11010.

A, B, D. Cross-sections of large pinnule showing the smooth abaxial side without trichomes. C, E–G. Cross-sections of small pinnule showing the smooth outer-facing side (=abaxial side of large pinnule) without trichomes. Slides: A–HY0886, B–HY0884, C–HY0884, D–HY0886, E–HY0884, F–HY0886, G–HY0885. Scales: 0.5 mm (A), 200 μm (B–G).

multiseriate and multicellular trichomes (Fig. S10F). The width of the small pinnules is largest in their middle part where it is up to 1.6 mm in specimen YNUPB11007 and up to 2 mm or more in specimen YNUPB11010, and tapers gradually toward the base and apex (Fig. S9; Fig. S10E–L). The apex of small pinnules is entire. The outer periclinal wall of the epidermal cells is thickened and even papillary in some places. Sclerenchyma cells in the mesophyll are usually distributed irregularly and do not gather in groups between veins near the outer-facing side of small pinnules (Fig. 3L; Fig. S10M–O; S13F, G) as in *Paratingia fuyuanensis* sp. nov. (Fig. 1I, J; Fig. S6G, H). In the middle and upper parts of small pinnules a mass of transfusion cells is located above the vein on the inner-facing side (Fig. 3L; Fig. S10M–O; Fig. S13F), but in the basal part of small pinnules transfusion tissue is weakly developed (Fig. 4H, I; Fig. S13C, G).

Large pinnules

Abaxial surface of the large pinnules are smooth and lack ribs (Fig. 3K; Fig. 4F, G, J; Fig. S8G; S13A, B, D). Thick multiseriate, multicellular trichomes as those seen in *Paratingia fuyuanensis* sp. nov. are only distributed on the abaxial surface of the pinnule base (Fig. 3D; Fig. S9D–F). The anatomical structures of large pinnules of the present specimens are similar to that of *Paratingia fuyuanensis* sp. nov. except that the transfusion tissue seems to be absent in the present specimens (Fig. 3K; Fig. 4F, G; Fig. S13B, D). Teeth on the lateral sides of large pinnules have not been observed.
